# Particles from the Echinococcus granulosus Laminated Layer Inhibit CD40 Upregulation in Dendritic Cells by Interfering with Akt Activation

**DOI:** 10.1128/IAI.00641-19

**Published:** 2019-11-18

**Authors:** Álvaro Pittini, Yamila E. Martínez-Acosta, Cecilia Casaravilla, Paula I. Seoane, Dominik Rückerl, Celia Quijano, Judith E. Allen, Álvaro Díaz

**Affiliations:** a Área Inmunología, Departamento de Biociencias (Facultad de Química) and Cátedra de Inmunología, Instituto de Química Biológica (Facultad de Ciencias), Universidad de la República, Montevideo, Uruguay; b Lydia Becker Institute for Immunology and Inflammation, Faculty of Biology, Medicine, and Health, School of Biological Sciences, University of Manchester, Manchester, United Kingdom; c Center for Free Radical and Biomedical Research (CEINBIO) and Departamento de Bioquímica (Facultad de Medicina), Universidad de la República, Montevideo, Uruguay; University of Pennsylvania

**Keywords:** AKT signaling, CD40, *Echinococcus*, PI3K, dendritic cells, helminths, membrane affinity-triggered signaling

## Abstract

The larval stage of the cestode *Echinococcus granulosus* causes cystic echinococcosis in humans and livestock. This larva is protected by the millimeter-thick, mucin-based laminated layer (LL), from which materials have to be shed to allow parasite growth. We previously reported that dendritic cells (DCs) respond to microscopic pieces of the mucin gel of the LL (pLL) with unconventional maturation phenotypes, in the absence or presence of Toll-like receptor (TLR) agonists, including lipopolysaccharide (LPS). We also reported that the presence of pLL inhibited the activating phosphorylation of the phosphatidylinositol 3-kinase (PI3K) effector Akt induced by granulocyte-macrophage colony-stimulating factor or interleukin-4. We now show that the inhibitory effect of pLL extends to LPS as a PI3K activator, and results in diminished phosphorylation of GSK3 downstream from Akt. Functionally, the inhibition of Akt and GSK3 phosphorylation are linked to the blunted upregulation of CD40, a major feature of the unconventional maturation phenotype. Paradoxically, all aspects of unconventional maturation induced by pLL depend on PI3K class I. Additional components of the phagocytic machinery are needed, but phagocytosis of pLL particles is not required. These observations hint at a DC response mechanism related to receptor-independent mechanisms proposed for certain crystalline and synthetic polymer-based particles; this would fit the previously reported lack of detection of molecular-level motifs necessary of the effects of pLL on DCs. Finally, we report that DCs exposed to pLL are able to condition DCs not exposed to the material so that these cannot upregulate CD40 in full in response to LPS.

## Abstract (precis)

The larval stage of the cestode *Echinococcus granulosus* causes cystic echinococcosis in humans and livestock. This larva is protected by the millimeter-thick, mucin-based laminated layer (LL), from which materials have to be shed to allow parasite growth. We previously reported that dendritic cells (DCs) respond to microscopic pieces of the mucin gel of the LL (pLL) with unconventional maturation phenotypes, in the absence or presence of Toll-like receptor (TLR) agonists, including lipopolysaccharide (LPS).

## INTRODUCTION

The laminated layer (LL) is the unique mucin-based acellular structure that protects the tissue-dwelling larval stages of flatworm parasites belonging to the genus *Echinococcus* (1–3). Within the genus, the most massive LL (up to 3 mm in thickness) is found in the Echinococcus granulosus species cluster, causative agent of cystic echinococcosis or hydatid disease in livestock species as well as humans (4, 5). Cystic echinococcosis is characterized by the growth within internal organ parenchymae of fluid-filled, bladder-like larvae (called hydatids) that can reach tens of centimeters in diameter. Each hydatid is defined by its hydatid wall, composed of a thin inner layer of cells (called the germinal layer) and the outer protective LL. The LL is essentially a meshwork of mucins giving rise to an aqueous gel; in *E. granulosus* specifically it additionally comprises dispersed nanodeposits of the calcium salt of inositol hexakisphosphate (2, 6–9). The mucin backbones comprise highly glycosylated domains and short nonglycosylated N-terminal extensions, while the mucin glycans are very rich in galactose (2, 3, 10–13).

The shedding of LL particles is an essential part of *Echinococcus* larval growth (2, 14). However, immune responses to the hydatid are usually blunted, with a distinctive lack of significant inflammatory infiltrates (15). Thus, we are interested in how dendritic cells (DCs) and other myeloid cells decode and respond to this unusual biological material. In order to explore this question we previously characterized *in vitro*-generated preparations of microscopic particles of the native mucin component of the LL (16). These preparations (termed pLL), which are handled as suspensions in physiological buffer or medium, comprise flexible and mostly flat-shaped gel particles of heterogenous sizes even after filtration. The preparations do not elicit any of a range of cytokines tested from bone marrow-derived dendritic cells (BMDCs) or bone marrow-derived macrophages (BMDMs) or *in vivo* after intraperitoneal injection into mice (16). In terms of surface markers, they do induce upregulation of CD86 and CD80, but not CD40 or MHC-II, in BMDCs and BMDMs. When tested in the same myeloid cell models in the context of costimulation with different Toll-like receptor (TLR) agonists, pLL causes enhancement of interleukin-10 (IL-10) and inhibition of IL-12p70 and IL-12/23p40 expression in a contact-dependent fashion (16). Furthermore, pLL enhances lipopolysaccharide (LPS)-induced CD86 expression but inhibits CD40 upregulation *in vitro* and *in vivo* (16). Thus, pLL gives rise to unconventional maturation phenotypes in myeloid cells, both in the absence and in the presence of TLR agonists.

Here, we address the intracellular signaling changes underlying the immunomodulating properties of pLL. We report that exposure to pLL inhibits the activation of the phosphatidylinositol 3-kinase (PI3K) effector Akt induced by the TLR4 agonist LPS, mechanistically connecting this inhibition to the previously observed blunting of TLR-driven CD40 upregulation.

## RESULTS

### The phosphorylation of Akt and GSK3 is inhibited in GM-CSF–BMDCs exposed to pLL.

**FIG 1:**
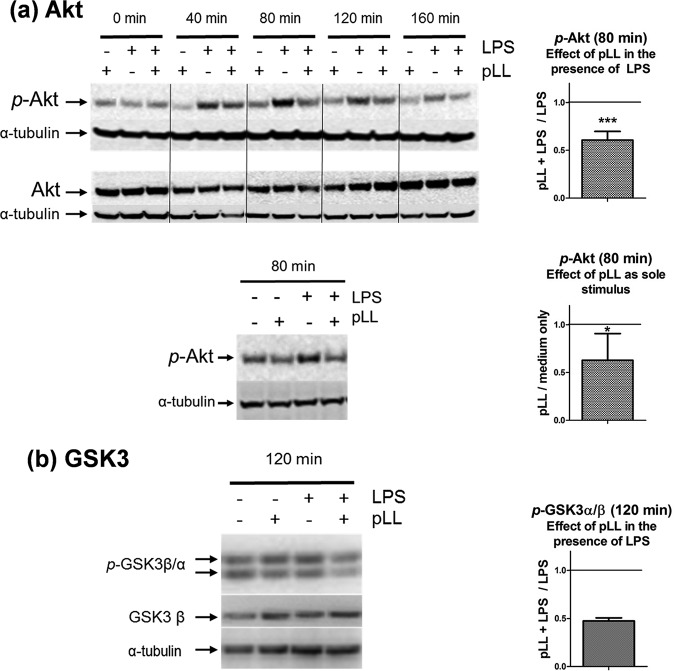
Exposure to pLL inhibits Akt (a) and GSK3 (b) phosphorylation in LPS-stimulated GM-CSF–BMDCs. GM-CSF–BMDCs were exposed to pLL, LPS, or both stimuli together for the indicated times. Cell lysates were analyzed for phosphorylated and total Akt (a) and for phosphorylated GSK3α and -β and total GSK3β (b). In panel a, the lower Western blot shows that pLL as the sole stimulus does not cause detectable Akt phosphorylation (similar results were obtained for incubation times between 10 and 160 min). The Western blots shown are representative of at least two independent experiments. The graphs show the quotients of p-Akt or p-GSK3α/β values (normalized over loading controls) for cells treated with pLL over cells not exposed to pLL in the presence or absence of LPS, as indicated. The graphs for p-Akt show means and standard deviations (SD) corresponding to six to eight independent experiments. Asterisks (*, *P* ≤ 0.05; ***, *P* ≤ 0.001) indicate significance as determined by a one-sample *t* test for comparison with unity (i.e., no inhibition). The graph for p-GSK3 shows the means and ranges of two independent experiments.

We previously reported that exposure of granulocyte-macrophage colony-stimulating factor (GM-CSF)–BMDCs to pLL blunts the (activating) Akt phosphorylation at Ser^473^ elicited by cytokine and growth factors that activate PI3K (17). TLR agonists, including LPS, also activate PI3K, and this pathway modulates DC activation, primarily driven by the NF-κB and mitogen-activated protein (MAP) kinase pathways (18–23) (see Fig. S1 in the supplemental material). Thus, in search of signaling changes underlying the unconventional maturation phenotypes elicited by pLL (16), we analyzed Akt phosphorylation in BMDCs stimulated with pLL and/or LPS. Exposure to pLL strongly inhibited Akt phosphorylation induced by LPS (Fig. 1a). As a sole stimulus, pLL did not cause detectable Akt phosphorylation; instead, it tended to inhibit basal phosphorylation (Fig. 1a), probably as a consequence of its capacity to inhibit Akt phosphorylation in response to GM-CSF, present in the culture medium (17). The effect on LPS-induced Akt phosphorylation was strongly diminished by disulfide reduction (Fig. S2), consistent with our previous finding that reduction of disulfide bonds in pLL, without altering its particulate nature, diminishes its effects on GM-CSF–BMDCs (16).

Akt directly phosphorylates GSK3 (24). In agreement with pLL inhibiting the activation of Akt, the phosphorylation of GSK3 was diminished in the presence of the particles (Fig. 1b).

In contrast to the effects on Akt and GSK3, exposure to pLL did not cause evident changes in the NF-κB and MAP kinase pathways, well known to impact conventional DC maturation in response to TLR agonists (25–32) (see Fig. S1a in the supplemental material). Specifically, the presence of pLL did not by itself cause degradation of IκB-α (Fig. S3), a necessary event in the activation of the canonical NF-κB pathway (32). This is in agreement with the previous observations that pLL does not elicit significant levels of any of several cytokines tested, from BMDCs or *in vivo* (16, 17). More importantly, upon costimulation with LPS, pLL did not alter canonical NF-κB activation (Fig. S3). The presence of pLL also did not alter the phosphorylation of the p38 or JNK MAP kinases induced by LPS (Fig. S4). pLL also did not appear to alter ERK phosphorylation, but our results were variable (data not shown). Since enhanced ERK activation is known to result in potentiated IL-10 output in myeloid cells (33), we carried out experiments in the presence of the MEK inhibitor UO126, which blocks ERK activation (34). Whereas the inhibitor as expected diminished IL-10 production, the effects of pLL on IL-10 and the remaining parameters measured were maintained in the presence of the inhibitor (Fig. S5).

In sum, the results of this section suggest that alterations in the PI3K pathway kinases Akt and GSK3, but not in the NF-κB or the MAP kinase pathways, are likely to underlie the observed ability of pLL to alter BMDC activation in response to LPS.

### Inhibited Akt and GSK3 phosphorylation is responsible for blunted CD40 upregulation in GM-CSF–BMDCs exposed to pLL.

**FIG 2:**
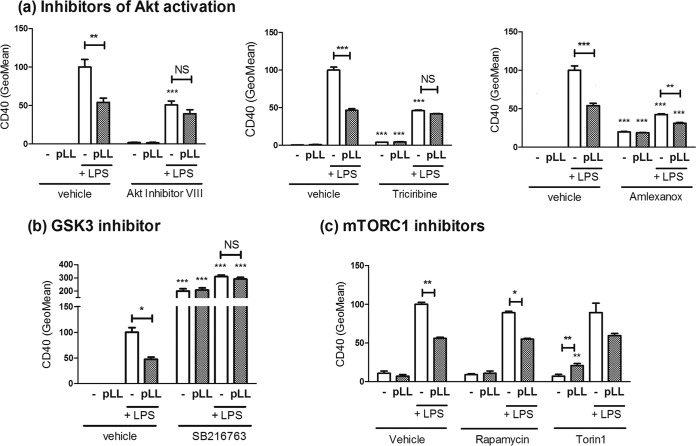
Akt inhibitors imitate and a GSK3 inhibitor reverses the blunted CD40 upregulation in the presence of pLL, whereas mTORC1 inhibitors are without effect. GM-CSF–BMDCs were exposed to pLL, LPS, or both stimuli together for 18 h in the absence or presence of inhibitors, and the surface expression of CD40 was measured. The inhibitors used interfere with Akt activation (Akt inhibitor VIII, triciribine, and amlexanox) (a), GSK3 activity (SB216763) (b), and mTORC1 activity (rapamycin and torin1) (c). Graphs show means ± the SD of triplicate wells. In the presence of torin1, the difference between LPS only and LPS+pLL is *P* = 0.052. The results shown are representative of two (Akt inhibitor VIII and triciribine) or three (amlexanox, SB261763, and mTORC1 inhibitors) independent experiments.

Both Akt and GSK3 are known to control the maturation status of myeloid cells (19, 21–23, 35–39). The participation of these kinases in the modulatory properties of pLL was tested with the help of three different inhibitors of Akt activation and an inhibitor of GSK3 activity. Two of the Akt inhibitors (Akt inhibitor VIII and triciribine) interfere with the recruitment of Akt to phosphoinositides in the plasma membrane necessary for activation, whereas the third (amlexanox) inhibits the kinases TBK-1 and IKK-ε, known to be necessary for LPS-induced Akt phosphorylation in BMDCs (40). As expected, the three Akt inhibitors abrogated LPS-induced Akt phosphorylation (data not shown). In contrast to the activating phosphorylation of Akt, the phosphorylation of GSK3 that we measured (at Ser^21^ of GSK3α and Ser^9^ of GSK3β, catalyzed by Akt) inhibits the kinase activity of GSK3 (24). If the previously observed effects of pLL were due to the observed alterations in Akt and GSK3 phosphorylation, the expectation would be that they are imitated by Akt inhibitors and are conversely negated by GSK3 inhibitors. This expectation was confirmed for the blunting of LPS-driven CD40 upregulation. Moreover, each of the three Akt inhibitors by itself blunted the upregulation of CD40 to an extent similar to pLL, and in the presence of the inhibitors exposure to pLL had either very weak or nil further effects (Fig. 2a). As for the GSK3 inhibitor, it enhanced CD40 induction in the presence of LPS, and in the presence of the inhibitor pLL did not blunt CD40 expression; in addition, the inhibitor caused strong CD40 expression in the absence of LPS (Fig. 2b).

In addition to phosphorylating GSK3 directly, Akt acts on GSK3 through the mTORC1 complex (22, 24). However, in contrast to the effects of the Akt and GSK3 inhibitors, two mTORC1 inhibitors had no effect on the LPS-driven induction of CD40, and in their presence pLL still blunted the upregulation of CD40 (Fig. 2c); the inhibitors nonetheless had the expected effects on cytokine output (see below).

In sum, exposure to pLL blunts CD40 upregulation because it inhibits Akt activity and thus enhances GSK3 activity, independently of any effects on mTORC1 (Fig. S6).

### The effects of pLL on CD86 and IL-10 are independent of alterations in Akt and GSK3 signaling.

**FIG 3:**
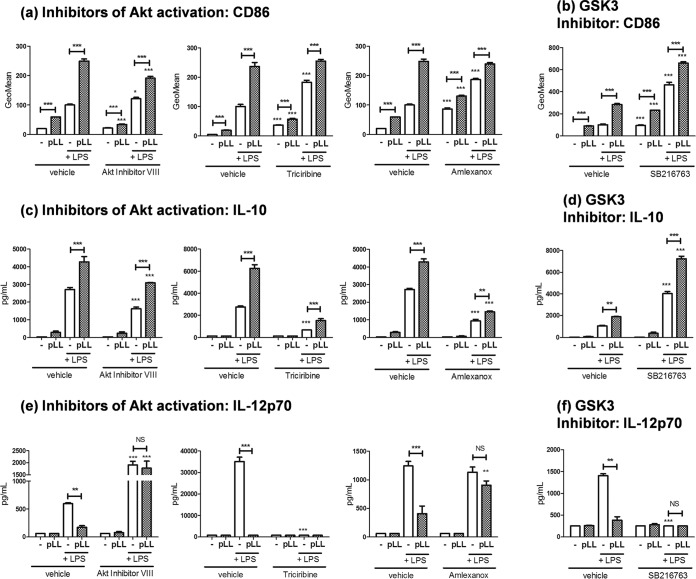
The changes induced by pLL in GM-CSF–BMDCs on CD86 and IL-10 expression are independent of alterations in Akt/GSK3 signaling. GM-CSF–BMDCs were exposed to pLL, LPS, or both stimuli together for 18 h in the absence or presence of inhibitors, and the cell surface expression of CD86 (a and b) and the levels of IL-10 (c and d) and IL-12p70 (e and f) in the supernatants were measured. The inhibitors used interfere with Akt activation (Akt inhibitor VIII, triciribine, and amlexanox) or GSK3 activity (SB216763). All data shown correspond to means ± the SD of triplicate wells. The results shown are representative of two (Akt inhibitor VIII and triciribine) or three (amlexanox and SB261763) independent experiments.

In contrast to the effects on CD40, the effects of pLL on CD86 and IL-10 could not be explained by the alterations of Akt and GSK3 phosphorylation. The enhancement of CD86 expression brought about by pLL, in the absence or presence of LPS, was still observed in the presence of the Akt or GSK3 inhibitors (Fig. 3a and b); independently of pLL, GSK3 inhibition enhanced CD86 expression, as expected (35). Similarly, the potentiating effect of pLL on IL-10 production was maintained in the presence of the inhibitors (Fig. 3c and d); as expected, Akt inhibitors reduced, whereas the GSK3 inhibitor enhanced, the IL-10 output of LPS-stimulated cells (19–23, 38).

The situation with IL-12p70 was more complex. On the one hand, no significant effects of pLL on the production of this cytokine were observed in the presence of the Akt or GSK3 inhibitors (Fig. 3e and f). On the other hand, compared to pLL, Akt inhibitors (except for triciribine) had the opposite effect on IL-12p70, whereas the GSK3 inhibitor had an effect in the same direction as pLL. The effects of the inhibitors (except for triciribine) on LPS-driven IL-12p70 production were in line with previously reported findings (19–23).

In the presence of the mTORC1 inhibitors, the effects of pLL on CD86, IL-10, and IL-12p70 were still observed; the inhibitors nonetheless had the expected effects on the production of IL-10 (inhibition) and IL-12p70 (enhancement) (Fig. S7) (19, 22).

Given these results, we hypothesized that the effects of pLL on CD86 and IL-10 (as well as possibly the effect on IL-12p70) may be due to alterations in the calcium/calcineurin/NFAT pathway (Fig. S1a), known to be important for myeloid cell responses to particulate stimuli (41, 42). However, neither the calcineurin inhibitor cyclosporine nor the extracellular calcium sequestering agent EGTA reversed the effects of pLL on CD86, IL-10, or IL-12p70 (Fig. S8a to c); the blunted CD40 upregulation was also unaffected by the treatments (Fig. S8d). In the absence of pLL, cyclosporine did not affect the response to LPS strongly, in agreement with precedents in which the inhibitor was also added to previously differentiated BMDCs (43, 44). However, at the higher dose used, the inhibitor decreased the IL-12p70 output, possibly consistent with the reported positive regulation of this cytokine by NFAT (45).

In sum, exposure of GM-CSF–BMDCs to pLL alters the expression of CD86 and IL-10 through unidentified signaling changes that are independent of the observed alterations in Akt and GSK3 activation (Fig. S6); also, these effects of pLL, as well as the remaining effects under study, appear to be independent of any changes in the calcium/calcineurin/NFAT pathway.

### In Flt3L-BMDCs, pLL inhibits neither Akt phosphorylation nor CD40 upregulation.

**FIG 4:**
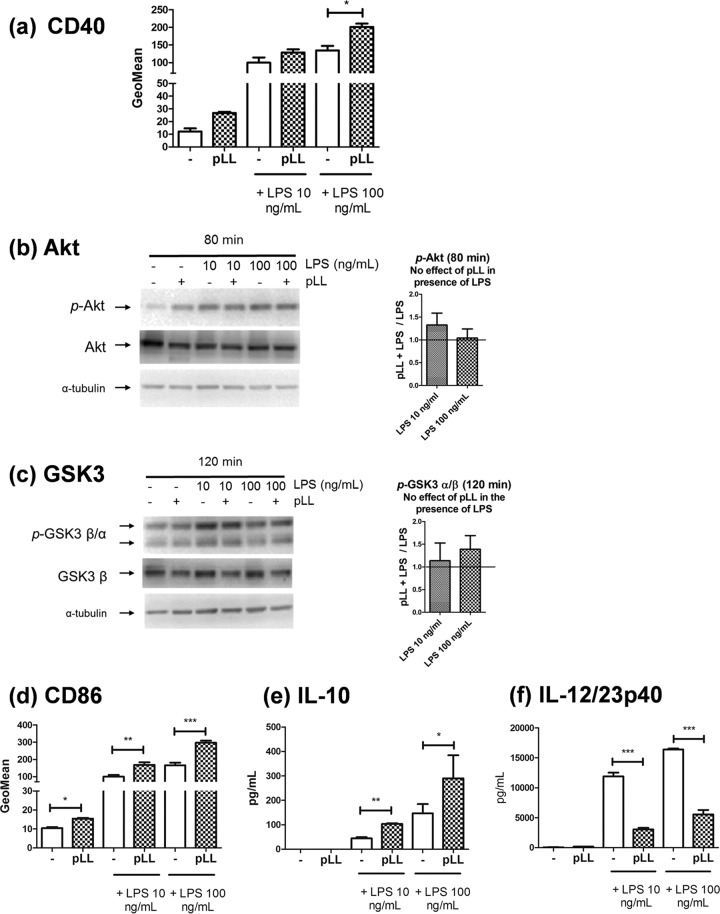
Neither Akt/GSK3 phosphorylation nor CD40 upregulation is blunted by pLL in Flt3L-BMDCs. Flt3L-BMDCs were exposed to pLL, LPS, or both stimuli together. After 18 h, the cells were analyzed for cell surface expression of CD40 and CD86 (a and d) or for IL-10 and IL-12/23p40 levels in supernatants (e and f). Alternatively, after the times indicated, cell lysates were generated for Western blot analysis (b and c); the graphs show of the quotients of p-Akt or p-GSK3a/b values (normalized over loading controls) for cells treated with pLL over cells not exposed to pLL (means and ranges of two independent experiments). Data plotted in panels a, d, e, and f represent means ± the SD of triplicate wells and are representative of at least two independent experiments; significant enhancement by pLL of CD40 expression induced by 100 ng/ml LPS was not reproduced across experiments.

Flt3L-BMDCs are a myeloid cell model proposed to represent splenic DCs (46, 47). We reasoned that analyzing the signaling and phenotypic changes under study in this second DC model may give us additional mechanistic information. In Flt3L-BMDCs, exposure to pLL did not inhibit CD40 upregulation induced by LPS and instead tended to enhance it (Fig. 4a). Notably, in Flt3L-BMDCs, the presence of pLL did not inhibit Akt or GSK3 phosphorylation (Fig. 4b and c). In contrast, modulatory effects of pLL on expression of CD86, IL-10 and IL-12/23p40 were still evident in Flt3L-BMDCs (Fig. 4d, e, and f).

In sum, pLL causes both inhibition of Akt phosphorylation and blunted CD40 upregulation in GM-CSF–BMDCs, whereas it causes neither effect in Flt3L-BMDCs. In contrast, effects of pLL on expression of CD86 and IL-10 can arise in the absence of effect on Akt phosphorylation. Both observations reinforce the conclusions drawn in the GM-CSF–BMDC model that blunted CD40 upregulation depends on inhibited Akt and GSK3 phosphorylation, whereas the effects on CD86 and IL-10 do not (Fig. S6).

### The effects of pLL on LPS-induced maturation of GM-CSF–BMDCs are not due to impaired switch to glycolytic metabolism.

**FIG 5:**
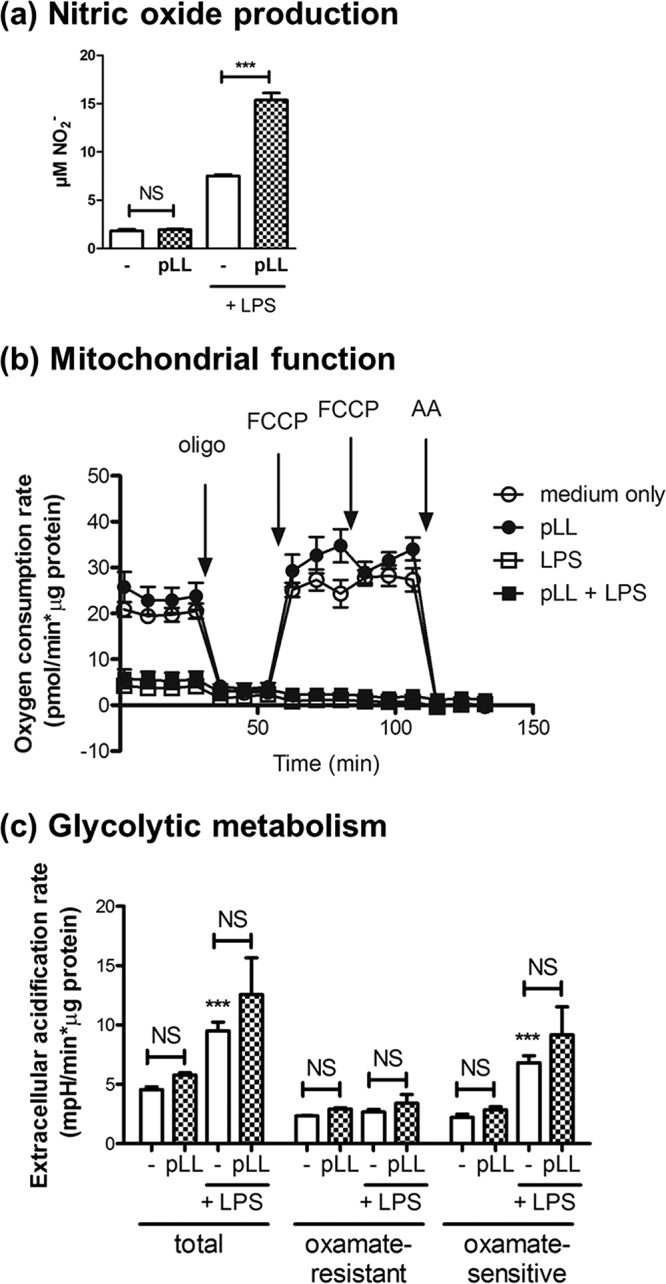
The phenotypic effects of pLL on GM-CSF–BMDCs are not due to an impaired switch to glycolytic metabolism. GM-CSF–BMDCs were exposed to pLL, LPS, or both stimuli together for 18 to 22 h; nitrite was measured in supernatants as an indication of **⋅**NO output (a) or mitochondrial function (b), and glycolytic fermentative metabolism was assessed with the help of a Seahorse XF-24 extracellular flux analyzer and appropriate inhibitors and/or uncoupling agents (c). Mitochondrial function was assessed by measuring the OCR before and after the sequential addition of the ATP synthase inhibitor oligomycin (oligo), the uncoupler FCCP, and the complex III inhibitor antimycin A (AA). Glycolytic fermentative metabolism was analyzed by measuring the extracellular acidification rate, specifically the portion of this rate that is sensitive to the lactate dehydrogenase inhibitor oxamate and therefore linked to lactate formation and excretion. The graphs show means ± the SD of three to four wells. The results shown are representative of at least two independent experiments.

In GM-CSF–BMDCs 8 to 10 h after LPS stimulation and onward, mitochondrial electron transport becomes blocked by the nitric oxide (⋅NO) produced by the cells (40, 48, 49). The cells then meet their energy demands via glycolytic fermentation, and inhibition of the switch to glycolysis impairs maturation, including upregulation of CD40 (49). Exposure to pLL did not inhibit, but actually enhanced, the LPS-driven ⋅NO production in GM-CSF–BMDCs (Fig. 5a). Of note, pLL by itself did not elicit ⋅NO synthesis, consistent with its lack of capacity to activate NF-κB or to elicit cytokines (16). In agreement with unimpaired ⋅NO generation, GM-CSF–BMDCs exposed to LPS plus pLL displayed fully blocked mitochondrial respiration, similar to cells treated with LPS alone. This included a decrease in basal respiration and unresponsiveness to inhibitors and uncoupler of oxidative phosphorylation (Fig. 5b and Table S1). These results made it important to assess whether GM-CSF–BMDCs treated with LPS plus pLL could upregulate glycolytic metabolism, a change that is known to depend on Akt (49). The presence of pLL did not inhibit the LPS-induced increase in extracellular acidification rate sensitive to the lactate dehydrogenase inhibitor oxamate, i.e., it did not inhibit the switch to a lactic fermentative metabolism (Fig. 5c). Thus, exposure of GM-CSF–BMDCs to pLL reverses neither the loss of mitochondrial respiration nor the accompanying switch to glycolytic fermentative metabolism that is induced by TLR agonists. This means that the unconventional maturation of GM-CSF–BMDCs in the presence of pLL is not associated with an impairment of the metabolic reprogramming brought about by TLR agonists.

### Immune modulation by pLL requires PI3K class I.

**FIG 6:**
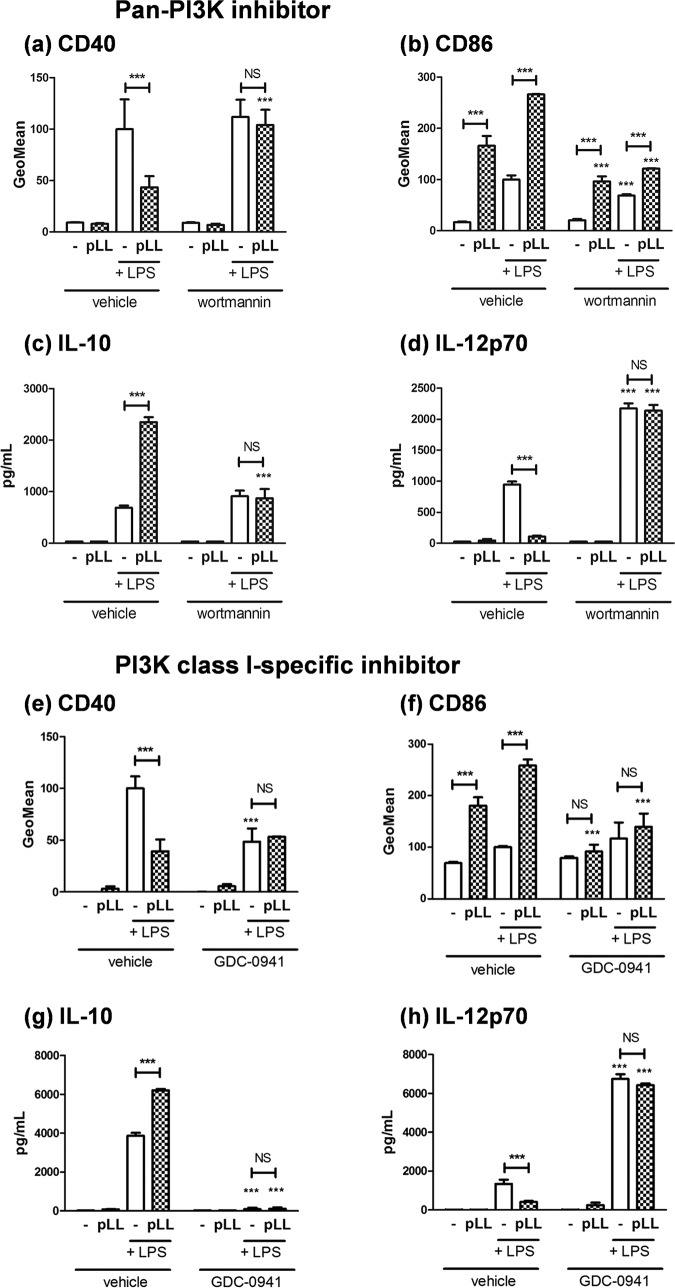
The phenotypic effects of pLL on GM-CSF–BMDCs require functional class I PI3Ks. GM-CSF–BMDCs were exposed to pLL, LPS, or both stimuli together for 18 h in the absence or presence of the pan-PI3K inhibitor wortmannin (a to d) or the PI3K class I-specific inhibitor GDC-0941 (e to h). The cell surface expression of CD40 (a and e) and CD86 (b and f) and the levels of IL-10 (c and g) and IL-12p70 (d and h) in supernatants were measured. All values plotted correspond to means ± the SD of triplicate wells. The results shown are representative of at least three independent experiments.

Since inhibition of PI3K strongly diminishes Akt and GSK3 phosphorylation in BMDCs (19), our expectation was that PI3K inhibitors would imitate the effect of pLL on CD40 expression. However, the pan-PI3K inhibitor wortmannin did not imitate the effect of pLL but instead abrogated the capacity of pLL to blunt CD40 upregulation (Fig. 6a). Interestingly, wortmannin strongly weakened the activity of pLL in terms of CD86 (Fig. 6b), and it abrogated pLL’s activity in terms of IL-10 and IL-12p70 (Fig. 6c and d). The PI3K class I-specific inhibitor GDC-0941 more clearly abrogated all of the effects of pLL under study (Fig. 6e to h), suggesting that PI3K class I is required for pLL to be active on BMDCs. In the absence of pLL, the PI3K-specific inhibitor blunted CD40 upregulation, consistent with PI3K-Akt activation being necessary for full CD40 upregulation. Also in the absence of pLL, the two PI3K inhibitors showed the expected effects on LPS-induced IL-12p70 and IL-10 responses (19, 20, 50).

The apparent incapacity of BMDCs to respond to pLL in the presence of PI3K inhibitors was similar to what was observed in the presence of the actin cytoskeleton inhibitor cytochalasin D (16). PI3K class I controls actin dynamics in myeloid cells in the contexts of phagocytosis and frustrated phagocytosis (51). An important player that collaborates with PI3K class I in these contexts is the kinase Syk. Analysis of the participation of Syk was made difficult because the inhibitor piceatannol by itself blunted CD40 upregulation (Fig. S9a) and abrogated IL-12p70 and IL-10 responses (data not shown) induced by LPS. This notwithstanding, no effect of pLL on CD40 and much weakened effects on CD86 were observed in the presence of the inhibitor (Fig. S9a and b), suggesting that Syk may participate in the effects of pLL.

In sum, the overall effects of pLL on GM-CSF–BMDC phenotype, already known to require actin dynamics, also require PI3K class I and may require Syk (Fig. S6).

### Immune modulation by pLL does not require particle phagocytosis.

**FIG 7:**
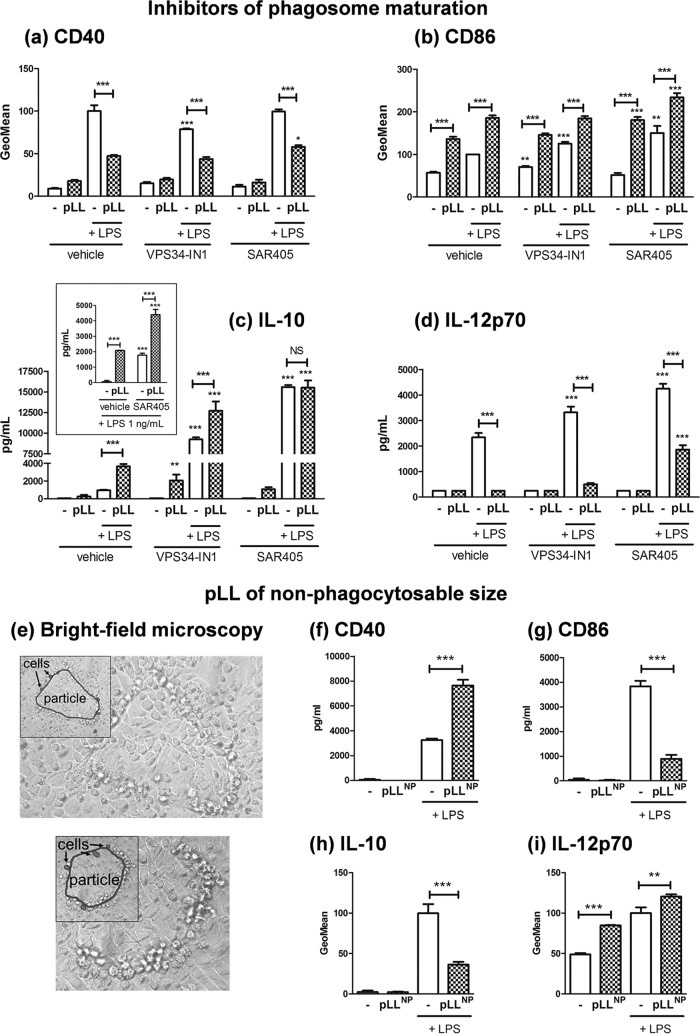
The phenotypic effects of pLL on GM-CSF–BMDCs do not require phagocytosis. GM-CSF–BMDCs were exposed to pLL, LPS (the usual 10 ng/ml or in the indicated case 1 ng/ml), or both stimuli together for 18 h in the absence or presence of inhibitors of PI3K class III (Vps34-IN1 and SAR405), and the cell surface expression of CD40 (a) and CD86 (b), as well as the levels of IL-10 (c) and IL-12p70 (d), in supernatants was measured. Also, GM-CSF–BMDCs were exposed to pLL selected for nonphagocytosable particle size range (“pLL^NP^”), LPS, or both stimuli together for 18 h, and maturation parameters were measured as described above. (e) Bright-field microscopy images of two representative nonphagocytosable pLL particles incubated with GM-CSF–BMDCs under assay conditions are shown. Insets highlight the contour of each particle (empty outline) and those of selected cells (outlines filled in gray). (f to i) Maturation parameters of GM-CSF–BMDCs responding to pLL^NP^ with or without LPS: CD40 (f) and CD86 (g) expression, as well as IL-10 (h) and IL-12p70 (i) production. All values plotted correspond to means ± the SD of triplicate wells. The results shown are representative of at least three independent experiments.

Given these results, the likeliest explanation was that the requirement for actin dynamics and PI3K class I reflected a need for particle internalization. The pLL preparation contains a range of particle dimensions encompassing both phagocytosable and nonphagocytosable sizes (16). However, we were unable to detect phagocytosis of fluorescently labeled pLL particles by GM-CSF–BMDCs by microscopy or flow cytometry (data not shown). Internalized particles are normally contained in phagosomes, the membrane of which recruits PI3K class III; the phosphoinositide product of this enzyme, PI(3)P, is a marker of late endosomes/phagosomes (51–53). If the effects of pLL under study were exerted from the phagosomal compartment, it is likely that they would be sensitive to inhibition of PI3K class III. However, all the effects of pLL were still observed in the presence of two well-characterized PI3K class III inhibitors, VPS34-IN1 and SAR405 (54, 55) (Fig. 7a to d). The two inhibitors caused the expected enhancement in LPS-driven production of cytokines (20, 56). In this context, the extremely high level of IL-10 elicited by LPS in the presence of one of the inhibitors (SAR405) was not further increased in the presence of pLL (Fig. 7c); however, by using a lower LPS dose (1 ng/ml) that elicits a more moderate IL-10 response, we confirmed that the effect of pLL in terms of IL-10 is intact in the presence of this inhibitor (Fig. 7c, inset). Thus, phagosome maturation is not necessary for the effects of pLL on GM-CSF–BMDCs.

We next tested a pLL preparation in which all particles were too large for phagocytosis. This preparation (nonphagocytosable pLL [pLL^NP^]; Fig. 7e) conditioned GM-CSF–BMDCs in the same ways previously described (Fig. 7f to i). Moreover, its effects were also abrogated by inhibition of actin dynamics or PI3K class I (Fig. S10).

In sum, the induction of the unconventional maturation phenotypes by pLL requires elements of the phagocytic machinery, but it can take place in the absence of phagocytosis.

### The blunting of CD40 upregulation caused by pLL can be transmitted to bystander DC, and this is independent of IL-10.

**FIG 8:**
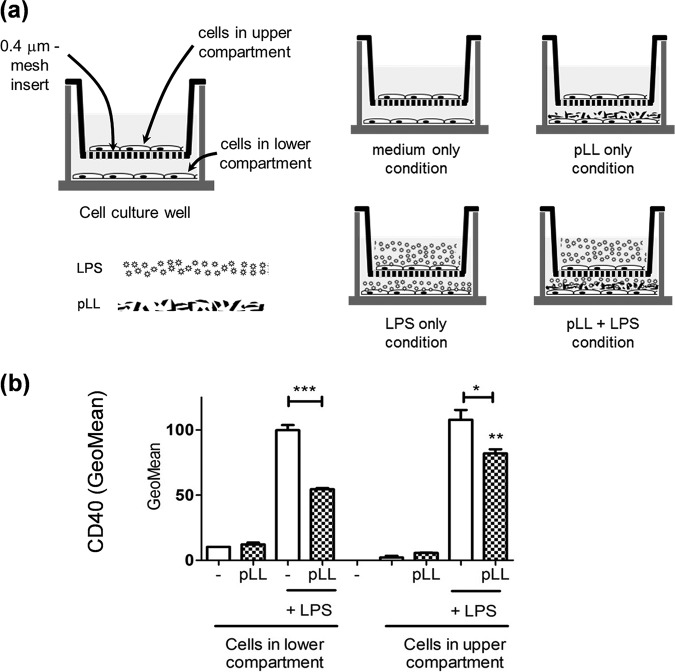
The diminished capacity to upregulate CD40 can be transmitted to cells not in contact with pLL. (a) GM-CSF–BMDCs were cultured for 18 h using permeable transwell inserts in the presence of pLL, LPS, or both stimuli; pLL when added was present in the lower compartment only, whereas LPS (when added) was present in both compartments, as depicted. (b) The cell surface expression of CD40 was then measured in cells from both compartments separately. This allowed us to assess the potential of cells in contact with pLL to condition cells not in contact with pLL via diffusible factors. The values plotted correspond to means ± the SD of triplicate wells. The results shown are representative of three independent experiments.

We reported previously that pLL present in the same well as BMDCs but separated from these by a transwell insert permeable to molecules does not interfere with CD40 upregulation (16). However, it was plausible that the conditioning for limited CD40 upregulation may be transmitted via diffusible factors from cells in contact with pLL to other cells not in contact with pLL. We therefore used the transwell insert system but now culturing cells in both compartments; pLL when added was present in the lower compartment only, whereas LPS when added was present in both compartments. We observed that BMDCs separated from pLL by the insert but present in the same wells as BMDCs in contact with pLL also had their CD40 response to LPS stimulation weakened (Fig. 8). Thus, cells in contact with pLL can condition other cells for blunted CD40 expression in a contact-independent manner.

These results suggest an Akt/GSK3-dependent effect of pLL on CD40 expression and parallel Akt/GSK3-independent effects on IL-10 (and CD86). However, IL-10 is known to inhibit LPS-induced CD40 upregulation in BMDCs (57), opening the possibility that the effects we observed on IL-10 and CD40 may be mechanistically connected. Blocking IL-10 in our system did not reverse the blunted CD40 upregulation (Fig. S11), even if it enhanced the IL-12p70 output, as expected (data not shown). The lack of effect on CD40 is probably due to IL-10 only reaching in our system approximately 5 ng/ml (in cells stimulated with pLL+LPS), in contrast to the treatment with 50 ng/ml exogenous IL-10 reported elsewhere (57). In sum, the presence of pLL can blunt CD40 upregulation in bystander DCs independently of effects on IL-10.

Overall, the data presented here indicate that the LL modulates TLR agonist-induced DC activation via partial inhibition of Akt activation and additional unidentified signaling changes, without gross interference with the NF-κB, MAPK, or NFAT pathways. This scenario is consistent with dampening of immune activation in the proximity of the parasite without causing overt immunosuppression and thus risking premature death of its host.

## DISCUSSION

The form in which DCs decode LL materials is important for the immunology of cystic echinococcosis and has a wider relevance for understanding innate responses to particles in general. Our previous reports on pLL modulatory activity (16, 17) suggested that it did not cause NF-κB activation but could modulate the ability of other mediators to activate this pathway. The results in the present paper support the absence of NF-κB activation by pLL and suggest that pLL conditions responses to TLR agonists by mechanisms that do not involve gross inhibition of NF-κB or the p38 or JNK MAP kinases, potentiation of the activation of the ERK MAP kinase, or gross alterations in calcium-NFAT signaling (see Fig. S3 to S5 and S8 in the supplemental material).

We found that exposure to pLL conditions responses to TLR agonists in GM-CSF–BMDCs in part through interference with Akt activation and consequently with the inactivating phosphorylation of GSK3 (Fig. 1 and 2a and b). The PI3K/Akt/GSK3 pathway is well known to modulate NF-κB-dependent responses in myeloid cells. PI3K/Akt activation and consequent GSK3 inactivation are considered anti-inflammatory because they restrict IL-12 and enhance IL-10 expression in myeloid cells including GM-CSF–BMDCs (18–20, 22, 23, 37–39, 50) (Fig. S1b). In contrast, PI3K/Akt activation and consequent GSK3 inactivation promote the expression of costimulatory molecules in GM-CSF–BMDCs and other myeloid cell models (21, 35, 36, 58); thus, the PI3K pathway generally promotes conventional DC maturation in terms of surface markers (Fig. S1b). In this context, only the blunting of CD40 expression by pLL is in line with its impact on Akt and GSK3 (hypophosphorylation of both and hence activation of GSK3). Our experiments with inhibitors and comparison with the Flt3L-BMDCs model fully supported a causal link between the effects of pLL on Akt and GSK3 phosphorylation and the limitation of CD40 induction in GM-CSF–BMDCs (Fig. 2 and 4 and Fig. S6 in the supplemental material). Akt phosphorylates GSK3 directly (24) but also acts on GSK3 through the mTORC1 complex (22). However, the effect of pLL on CD40 in GM-CSF–BMDCs was independent of mTORC1 (Fig. 2c). Our conclusion that pLL blunts CD40 upregulation by altering Akt and GSK3 activation is in line with previous reports that Akt activation is necessary for full induction of CD40 and that GSK3 activity negatively controls the expression of the same marker in GM-CSF–BMDCs stimulated with LPS and/or other maturation signals (35, 36, 58).

In contrast to the situation with CD40, the impact of pLL on CD86 (and CD80 [16]) and IL-10 is at odds with its effects on Akt and GSK3 phosphorylation. Inhibitor data and the Flt3L-BMDC model (Fig. 3 and 4) support the conclusion that exposure to pLL affects CD86 and IL-10 expression independently of the changes in Akt and GSK3 and probably in spite of these changes (Fig. S6). A model that includes parallel signaling alterations in Akt/GSK3 (leading to interference with CD40 upregulation) and alterations in unidentified signaling components (leading to the alterations in CD86 and IL-10 expression) is consistent with the observation that the observed effect on CD40 is independent of the potentiation of IL-10 (Fig. S11). With respect to the inhibition of IL-12, the data did not allow us to conclude whether it is mediated by Akt/GSK3-dependent or -independent mechanisms or by a combination of both.

Although Akt activation is needed for the long-term switch to glycolysis by TLR-activated GM-CSF–BMDCs (49), this switch was not inhibited by pLL (Fig. 5b and c). This may be because the presence of pLL blunts LPS-driven Akt activation but does not fully inhibit it (Fig. 2), as previously observed in response to other PI3K-activating stimuli (17). The ⋅NO production that is known to bring about the glycolytic switch in LPS-stimulated BMDCs was actually augmented by pLL (Fig. 5a). This may be a consequence of the defective GSK3 phospho-inactivation observed, since GSK3 is known to positively regulate inducible nitric oxide synthase (iNOS) expression and ⋅NO output (37, 59, 60). Our results on the effect of pLL on ⋅NO production in GM-CSF–BMDCs contrast somewhat with published results, obtained in peritoneal macrophages (61, 62). These differences likely arise from fundamental differences between preparations with regard to components not intrinsic to the LL (63).

The overall ability of pLL to alter BMDC activation failed or was strongly weakened in the presence of PI3K inhibitors (Fig. 6). A similar shutdown of BMDC responses to pLL was previously observed in the presence of an actin polymerization inhibitor (16). Also, a similar, although less clear, result was obtained in the presence of a Syk inhibitor (Fig. S9). PI3K class I, the actin cytoskeleton, and Syk are part of the phagocytic machinery. However, our data indicated that phagocytosis is not indispensable for pLL to be active (Fig. 7), even if we cannot rule out a quantitative contribution from possible internalized pLL particles. The elicitation of myeloid cell responses by insoluble materials in a manner dependent on PI3K class I, Syk, and the actin cytoskeleton without a requirement for particle internalization is the hallmark of “membrane affinity triggered signaling” (MATS) (64–68). This mechanism is put forward to explain myeloid cell responses to crystalline or synthetic polymer-based materials. It posits that the interaction between solid surfaces and plasma membrane lipids triggers a receptor-independent form of ITAM-Syk signaling (66, 68). The requirement for PI3K and actin dynamics in addition to Syk is rationalized on the basis of the cells having to spread on the solid surfaces to generate interactions sufficiently strong for the unconventional signaling; phagocytosis can ensue, but it is not a requirement for signaling (65, 68). Thus, in terms of mechanistic requirements, MATS fits our observations with pLL better than does conventional pattern recognition receptor (PRR)-initiated signaling: even with PRR agonists that require particulate presentation, as exemplified by β1-3 glucan particles (acting via dectin-1), responses are modulated (positively or negatively) but not abrogated by PI3K or actin inhibitors (69–73). A receptor-independent mechanism also fits our observations because we have failed to identify molecular-level motifs in pLL that are needed for BMDC responses. Indeed, pLL subjected to carbohydrate oxidation or proteolysis is still active (16), while the soluble or solubilized and plate-adsorbed LL mucins are not (unpublished results). Since disulfide reduction weakens the effects of pLL (16) (Fig. S2) and mucins may be partly protected from proteolysis, the possibility remained that protein recognition contributed to the response. However, the N terminus of the apomucin EgrG_000742900.1 bears the only cysteine residue in the predicted LL apomucins (3, 11) and the corresponding synthetic peptide (sequence HACKQSPPPM; peptide dimerized through a disulfide bond and amidated at the C terminus) had no effect on BMDCs either as an agonist or as a pLL antagonist (unpublished results). It is therefore more likely that disulfide reduction affects material-level properties of LL particles (7). The previous idea does not exclude that conventional recognition/response mechanisms may participate in host responses to the LL. For example, the lectin CLEC4F, a Kupffer cell-specific protein in rodents (74), is known to bind the LL carbohydrates (3, 75).

Our previous report (17), together with the present results (Fig. 1), reveals that pLL interferes with Akt activation downstream of disparate receptors that couple to PI3K. MATS signaling is now known to be based on the redistribution of plasma membrane phosphatidylinositol 4,5-bisphosphate (PIP2) to the area of interaction with particles and consequent PIP2 binding by the abundant cytosolic (ITAM-containing) protein moesin (68). We speculate that this process can decrease the activity of receptor-activated PI3K class I through diminished availability of its PIP2 substrate, especially in the context of a prolonged interaction at the cell surface, as established by pLL. In agreement, PIP2 availability regulates PI3K activity in some contexts (76, 77). Thus, a MATS mechanism could explain why GM-CSF–BMDC responses to pLL require PI3K and yet result in blunting of Akt activation in response to other stimuli (Fig. S6).

The modulatory effects of pLL are likely induced *in vivo* not only by shed LL particles but also by the surface of the LL itself, as suggested by our results using nonphagocytosable pLL (Fig. 7). Of these effects, the blunting of CD40 upregulation in particular may not affect all DC subtypes, as suggested by our contrasting results with the GM-CSF–BMDC and Flt3L-BMDC models (Fig. 1, 2, and 4). The effect does occur *in vivo*, as shown by analysis of peritoneal cavity DCs after LPS and pLL injection (16). In the infection context, interference with CD40 upregulation can be expected to affect in particular inflammatory DCs, for which GM-CSF–BMDCs are a model (47). The effect could be propagated to those inflammatory DCs in the parasite’s vicinity that are not in contact with either the LL or shed LL particles, as suggested by our results using transwell inserts (Fig. 8). A plausible mechanism for this propagation is the export of active GSK3 in extracellular vesicles; myeloid cells stimulated with TLR agonists are known to share their cytosolic contents with other cells via such vesicles (78, 79). Interference with CD40 upregulation in inflammatory DCs could impact the adaptive response to the infection, as CD40 participates in the induction of Th1 and Th2 responses against helminth infections (80, 81).

Exploration of the host response to metazoan parasites continues to be a rich source of new immunological knowledge (82–84). Our study uncovers a mechanism of interference with DC maturation not previously documented for a pathogen. It also suggests that innate detection of pathogen-derived insoluble materials can use mechanisms related to those that sense inorganic and synthetic particles, in addition to employing PRRs.

## MATERIALS AND METHODS

### Parasite materials.

pLL was generated as described, the dehydration step being carried out by freeze-drying (16). Nonphagocytosable pLL (pLL^NP^) was prepared by a variation of the basic protocol in which the particles retrieved were those retained in the 23-μm gauze instead of those passing this gauze; a previous filtration step through a 45-μm-pore-size filter was also added to the basic protocol to exclude very large particles. Note that pLL particles, being flexible, tend to pass through gauze that has a finer mesh that the longest dimension of the particles (16). In agreement, particles in the pLL^NP^ preparation invariably had sizes well in excess of 23 μm. The pLL and pLL^NP^ preparations had their concentrations determined and were stored as previously described (16). pLL preparations tested negative for endotoxin by the *Limulus* amebocyte lysate method (16).

### Chemical inhibitors.

Chemical inhibitors used were cytochalasin D (5 μM), amlexanox (200 μM), and wortmannin (100 nM) (all 3 from Sigma-Aldrich); UO126 (5 μM), rapamycin (10 nM), and Torin1 (10 nM) (all from Cell Signaling Technology); GDC-0941 (5 μM) and SB216763 (10 μM) (both from APExBIO); triciribine (10 μM; Calbiochem/Thermo); Akt inhibitor VIII (10 μM; Merck/Millipore); cyclosporine (10 and 50 ng/ml) and EGTA (2 mM; both from Sigma); piceatannol (25 μM; Santa Cruz Biotechnology); and VPS34-IN1 (1 μM) and SAR405 (1 μM) (both from the Division of Signal Transduction Therapy Unit, University of Dundee).

### BMDC generation.

GM-CSF–BMDCs were generated from C57BL/6 mice as described previously (16). Flt3L-BMDCs from the same mouse strain were generated by differentiation during 9 days in the presence of 200 ng/ml recombinant Ftl3L (PeproTech) as described previously (85, 86). BMDCs obtained were approximately 90% CD11c^+^.

### BMDC stimulation and measurement of cell responses.

GM-CSF–BMDCs were stimulated with pLL (25 μg total dry mass per million cells)/pLL^NP^ (75 μg total dry mass per million cells) and/or LPS (from Escherichia coli O127:B8; Sigma; 10 ng/ml) as described previously (16). pLL/pLL^NP^ and LPS were added at the same time. Any inhibitors used (except in the case of Seahorse experiments) were added to the cells 30 min before the addition of pLL/pLL^NP^ and/or LPS; control wells were added to corresponding concentrations of the vehicle used to dissolve the inhibitor(s). The inhibitor and vehicle doses used were previously verified not to affect cell viability significantly by To-Pro3 exclusion in flow cytometry. Flt3L-BMDCs were stimulated similarly to GM-CSF–BMDCs but in medium containing 50 ng/ml Flt3L, using a pLL dose of 50 μg total dry mass per million cells and LPS concentrations of either 10 or 100 ng/ml, as indicated. Cell surface markers and cytokines in supernatants were measured as reported previously (16); here, CD40 and CD86 levels are expressed in terms of the geometric mean fluorescence intensity normalized over the mean values for cells treated with 10 ng/ml LPS only. Nitrite (indicative of ⋅NO production) was measured by the Griess method, as reported by Seoane et al. (17). IL-10 blockade was carried out using the JES5-16E3 antibody or an isotype-matched control (both at 1 mg/ml; Thermo). In some experiments, cells were cultured in 24-well plates with Corning polycarbonate membrane inserts (pore size, 0.4 μm); 2 × 10^6^ and 0.34 × 10^6^ cells were present in the lower and upper well compartments, respectively, which corresponds to similar cell densities. In these experiments pLL particles were added to the lower compartment only (at 25 μg total dry mass per million cells), whereas LPS (10 ng/ml) was added to both compartments.

### Measurement of extracellular acidification and oxygen consumption rates.

Measurements were carried out simultaneously in a Seahorse XFe24 extracellular flux analyzer (Agilent). GM-CSF–BMDCs were plated in XFe24 plates at 200,000 cells per well in 100 μl. Then, pLL and/or LPS was added at the doses previously specified, completing 200-μl volumes per well. After overnight stimulation (18 to 22 h) under usual culture conditions, the cells were washed twice in nonbuffered medium (RPMI, 10% fetal bovine serum [FBS], and 5 ng/ml GM-CSF [pH 7.4]), and 600 μl of the same medium was added to each well. Cells were incubated for 1 h at 37°C in the absence of a CO_2_ atmosphere.

For mitochondrial respiratory analysis, basal oxygen consumption rate (OCR) measurements were made before the injection of inhibitors or the uncoupler. Successive measurements were then made after the sequential addition of an ATP-synthase inhibitor (oligomycin, 1 μM), an uncoupler [carbonyl cyanide-4-(trifluoromethoxy) phenylhydrazine (FCCP), 2 μM, two additions], and a complex III inhibitor (antimycin A, 1.7 μM). The nonmitochondrial OCR was determined after adding antimycin A and subtracted from all other values before calculating the respiratory parameters. The ATP-independent OCR was determined after oligomycin injection. The ATP-dependent OCR (respiration driving ATP synthesis) was calculated as the difference between basal and ATP-independent OCR. The maximum OCR was the maximal rate measured after FCCP injection (87).

To assess glucose fermentation to lactate, the extracellular acidification rate (ECAR) was measured before and after the addition of oxamate (50 mM), an inhibitor of lactate dehydrogenase (88). Basal measurements were taken before the injection of oxamate; oxamate-resistant ECAR was measured after the addition of the inhibitor, and the oxamate-sensitive ECAR was calculated as the difference between the previous two parameters.

For both OCR and ECAR, four baseline measurements and three response rates (after the addition of each compound) were obtained; the averages of these measurements, calculated for each of three independent wells for each condition, were used for data analysis.

### Antibodies and Western blotting.

The antibodies used in Western blotting are listed in Table S2 in the supplemental material. Cell lysates were prepared, and Western blots were carried out and analyzed as described previously (17), except that housekeeping proteins detected with the help of specific antibodies were in some cases used as loading controls.

### Statistics.

Except in one indicated case, quantitative results were analyzed by one-way analysis of variance, with a Tukey posttest. Data for CD40 and CD86 expression levels were transformed before analysis (by square root and logarithm, respectively) in order to remove skew and/or homogenize the variances corresponding to the different conditions. Significant differences are indicated by asterisks (*, *P* < 0.05; **, *P* < 0.01; ***, *P* < 0.001), and asterisks not associated with connecting lines represent differences with respect to cells stimulated in the same manner except for the absence of inhibitor being tested.

## Supplementary Material

Supplemental file 1
