# Supplementary material for: Particles from the Echinococcus granulosus Laminated Layer Inhibit CD40 Upregulation in Dendritic Cells by Interfering with Akt Activation
Source: Infect Immun. 2019 Nov 18;87(12):e00641-19. doi: 10.1128/IAI.00641-19 (PMC6867849; doi:10.1128/IAI.00641-19)
Supplement: Supplemental file 1 [file IAI.00641-19-s0001.pdf]

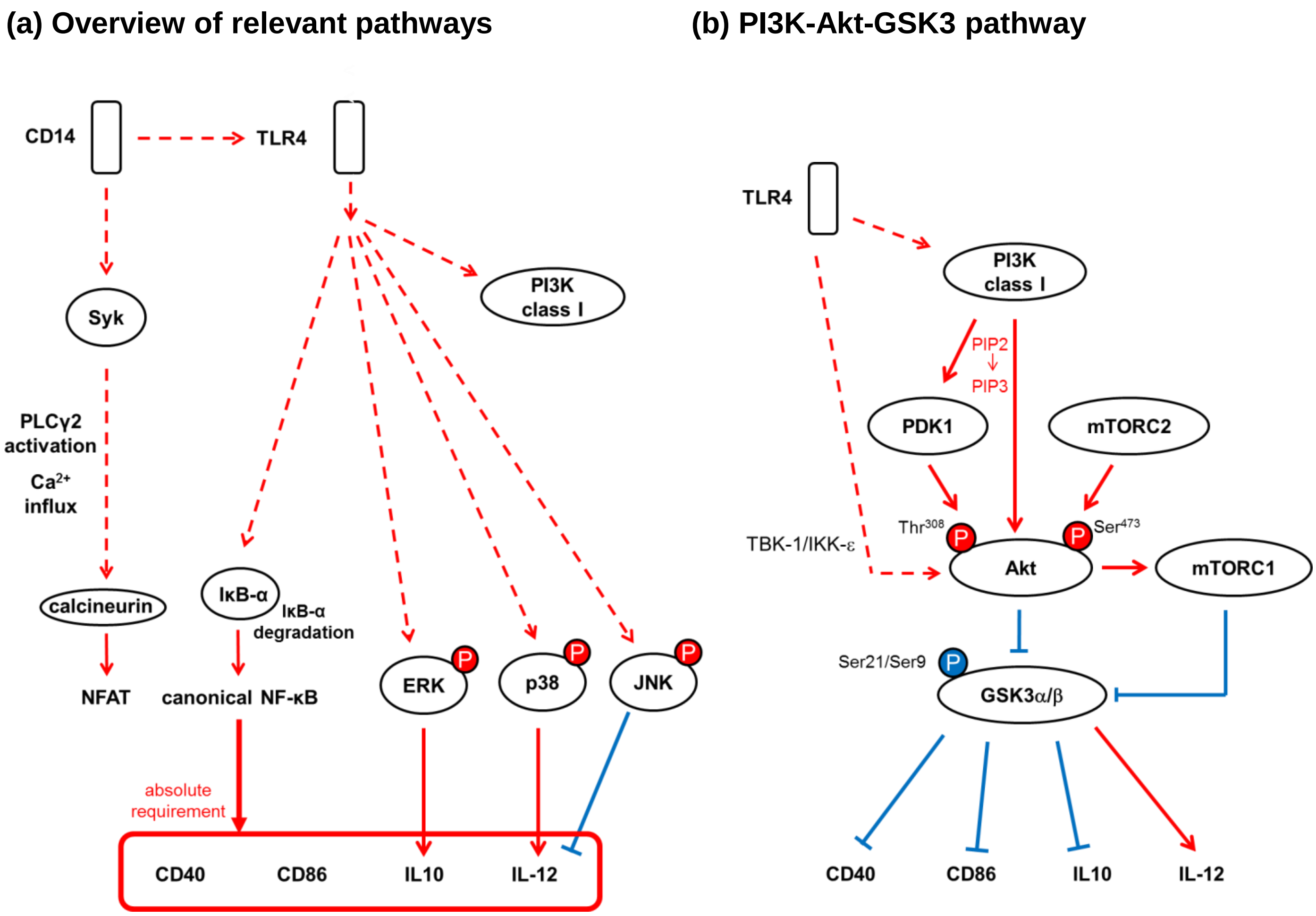

FIG S1. Summary of the known participation of the NF-κB, MAP kinases, Ca^{2+}-calcineurin-NFAT and PI3K-Akt-GSK3 pathwasy in the activation of GMCSF-BMDCs and other myeloid cell models in response to LPS. (See full legend on the next page).

FIG S1. Summary of the known participation of the NF-κB, MAP kinases, Ca^{2+}-calcineurin-NFAT and PI3K-Akt-GSK3 pathwasy in the activation of GMCSF-BMDCs and other myeloid cell models in response to LPS.

For clarity, a general view of the pathways is presented in **(a)** and the PI3K-Akt-GSK3 pathway is detailed in **(b)**. Only major points relevant to the present paper are shown. Red and blue lines represent activating and inhibitory effects respectively. Similarly, red and blue circles represent activating and inhibitory phosphorylation events respectively, and only phosphorylation events analyzed in the paper are represented.

LPS is mainly sensed by TLR4, in cooperation with CD14. TLR4 activation leads, through a complex series of steps, to the largely parallel activation (with certain early steps in common) of each of the MAP kinases (p38, JNK and ERK) and of the canonical NF-κB pathway. The central step in canonical NF-κB activation is the proteolytic degradation of IκB-α, which releases active NF-κB subunits for their nuclear translocation. Canonical NF-κB activation is required for up-regulation of the co-stimulatory molecules CD86 and CD40 and for expression of the cytokines IL-10 and IL-12. The p38 MAP kinase is needed for full IL-12 expression; contradictory data are available about its effects on CD86, CD40 and IL-10. JNK downregulates IL-12 expression whereas its effects on CD86, CD40 and IL-10 are not well established. ERK is dispensable for CD86 expression, whereas its effects on CD40 expression are not established; ERK is needed for IL-10 expression and dispensable for IL-12 expression.

Also in response to LPS, CD14 initiates, independently of TLR4 and dependent on Syk and phospholipase Cγ2 (PLCγ2), Ca^{2+} influx leading to calcineurin activation and therefore the activating dephosphorylation of NFAT. Information on the role of the NFAT pathway on expression of CD86, CD40, IL-10 and IL-12 in LPS-stimulated DCs is lacking or contradictory.

TLR4 activation additionally leads to activation of PI3K, through complex steps that are omitted for simplicity. PI3K catalyzes the conversion of phosphatidylinositol(4,5) bisphosphate (PIP2) into phosphatidylinositol(3,4,5)trisphosphate (PIP3). PIP3 promotes the recruitment of PDK1 and Akt to the plasma membrane, facilitating the activation by phosphorylation of both these kinases. Active PDK1 and the mTORC2 complex doubly phosphorylate Akt, causing its full activation. The kinases TBK-1 and IKK-ε are also required for LPS-driven Akt phosphorylation in BMDCs, but the exact mechanism by which they act is unclear. Akt directly phosphorylates GSK3, inactivating this kinase. Akt additionally, and indirectly, activates the mTORC1 complex, which also leads to GSK3 phosphorylation through the p70S6K kinase. Active GSK3 has negative effects on CD86 and CD40 and IL-10 expression, and a positive effect on IL-12 expression. Thus, activation of the PI3K pathway enhances the expression of CD40, CD86 and IL-10, through a "double-negative" (inhibition-inhibition) mechanism; in contrast, activation of the pathway inhibits expression of IL-12.

The diagram is based on references (1-22); see "References in Supplementary Materials", at the end of this document.

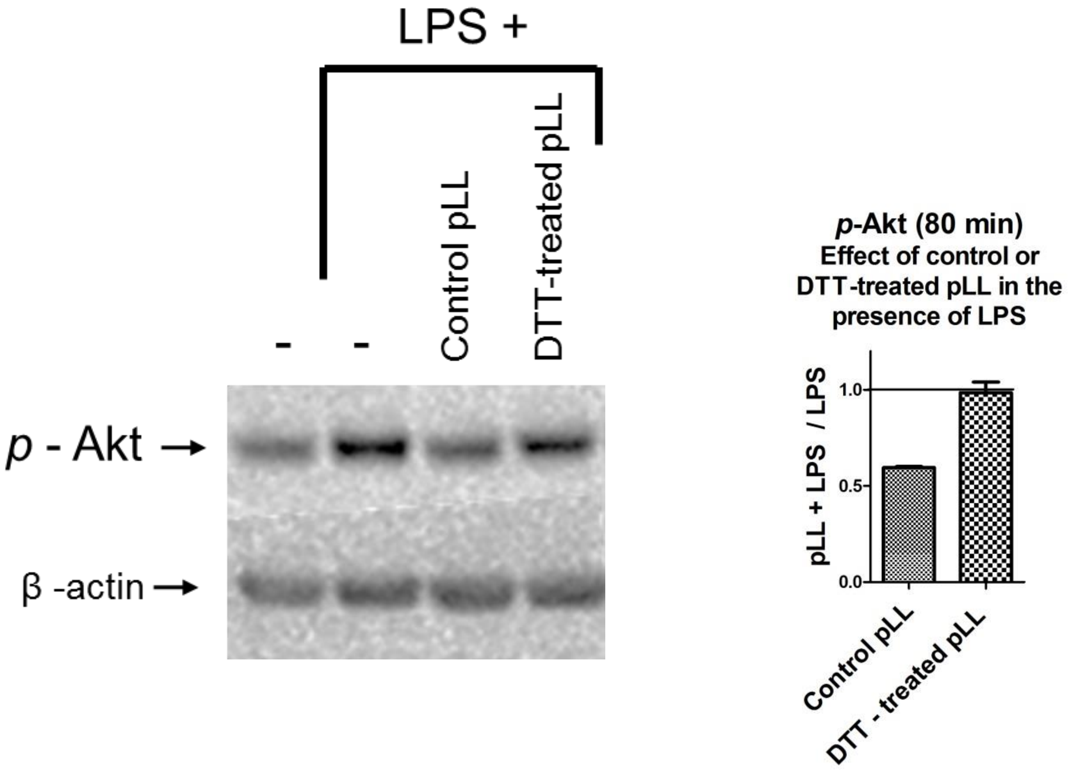

FIG S2 (related to Fig. 1). Reduction of disulphides in pLL abrogates its capacity to inhibit Akt phosphorylation in GMCSF-BMDCs. GMCSF-BMDCs were exposed to medium only, LPS, or LPS together with either pLL or pLL previously treated with DTT for disulfide reduction. After 80 min, cell lysates were prepared, and subsequently analyzed for phosphorylated Akt. The result shown is representative of 2 independent experiments. The graphs show the quotients of p-Akt values (normalized over loading controls) for cells treated with pLL (either control or DTT-treated) plus LPS over cells exposed to LPS only; values shown are mean and range of the 2 independent experiments.

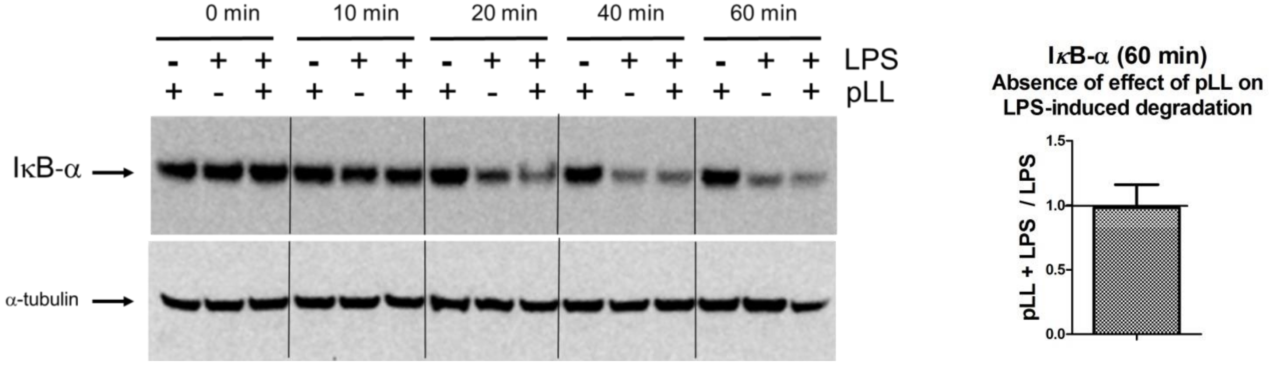

FIG S3. The effects of pLL on GMCSF-BMDCs are not associated with gross alteration in the activation of the canonical NF-κB pathway. GMCSF-BMDCs were exposed to pLL, LPS, or both stimuli together, for the indicated times. Cell lysates were analyzed for total IκB-α. The Western blot shown is representative of 2 independent experiments. The graph shows the quotients of the IκB-α amounts (normalized over loading controls) for cells treated with pLL plus LPS over cells exposed to LPS only, for the 60-min timepoint; values are the mean and range of the 2 independent experiments.

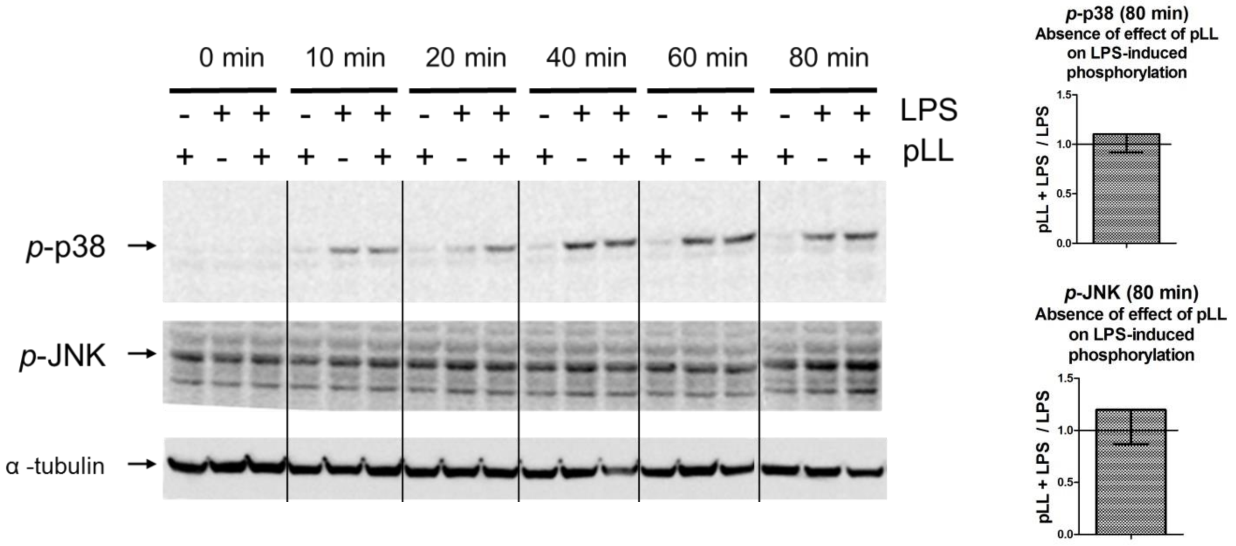

FIG S4. The effects of pLL on GMCSF-BMDCs are not associated with gross alterations in the activation of the p38 or JNK MAP kinases. GMCSF-BMDCs were exposed to pLL, LPS, or both stimuli together, for the indicated times. Cell lysates were analyzed for phosphorylated p38 and JNK MAP kinases. Results shown are representative of 3 independent experiments. The graphs show the quotients of *p*-p38 or *p*-JNK values respectively (normalized over loading controls) for cells treated with pLL plus LPS over cells treated with LPS only, for the 80-min timepoint; values shown are the mean and SD of the 3 independent experiments.

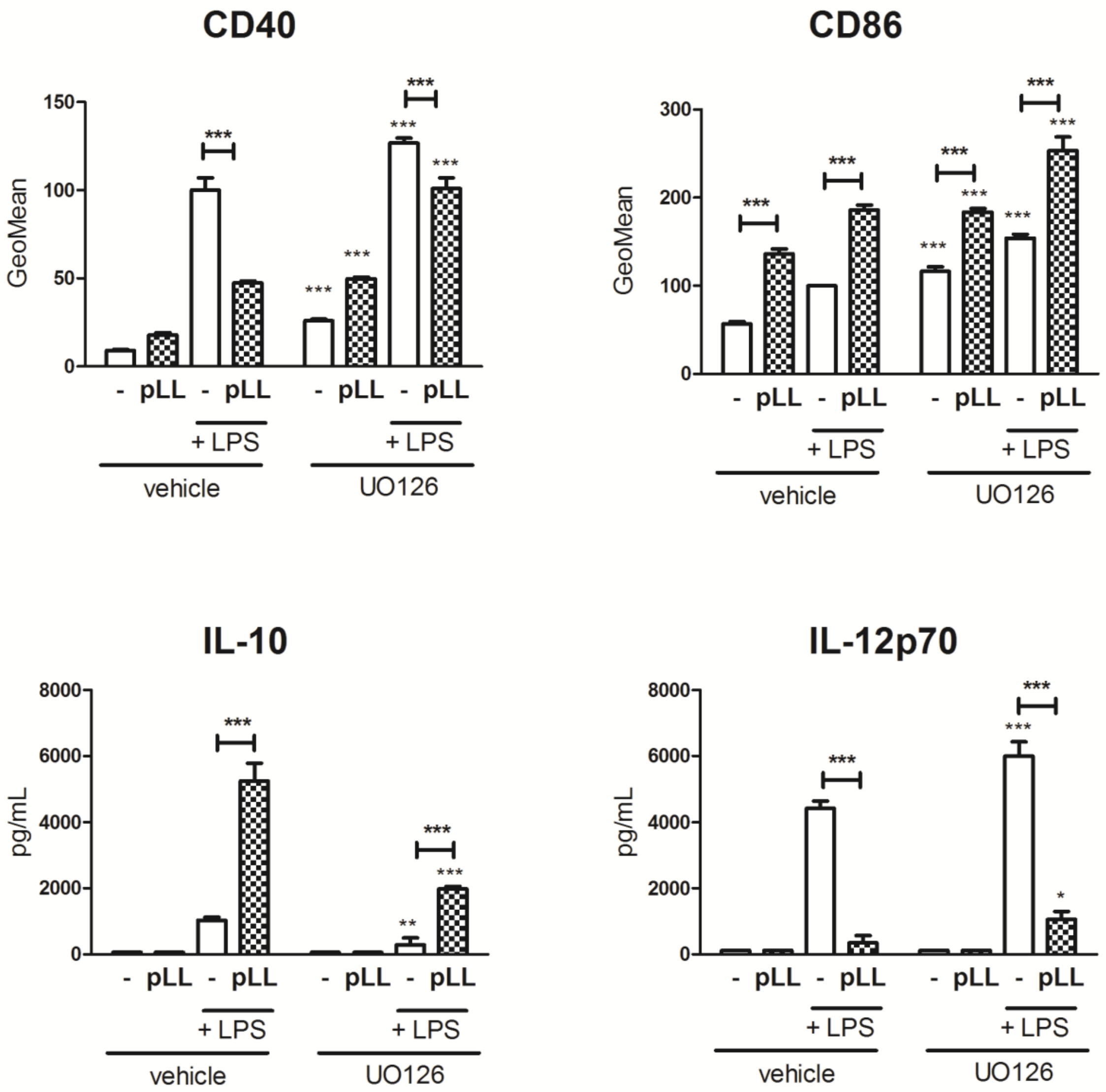

FIG S5. The effects of pLL on GMCSF-BMDCs are not abrogated by inhibition of ERK. GMCSF-BMDCs were exposed to pLL, LPS, or both stimuli together, for 18 h, in the absence or presence of the ERK activation inhibitor UO126. Cell surface expression of CD40 and CD86 and levels of IL-10 and IL-12p70 in supernatants were measured. All values plotted correspond to means +/- SD of triplicate wells. Results shown are representative of 2 independent experiments.

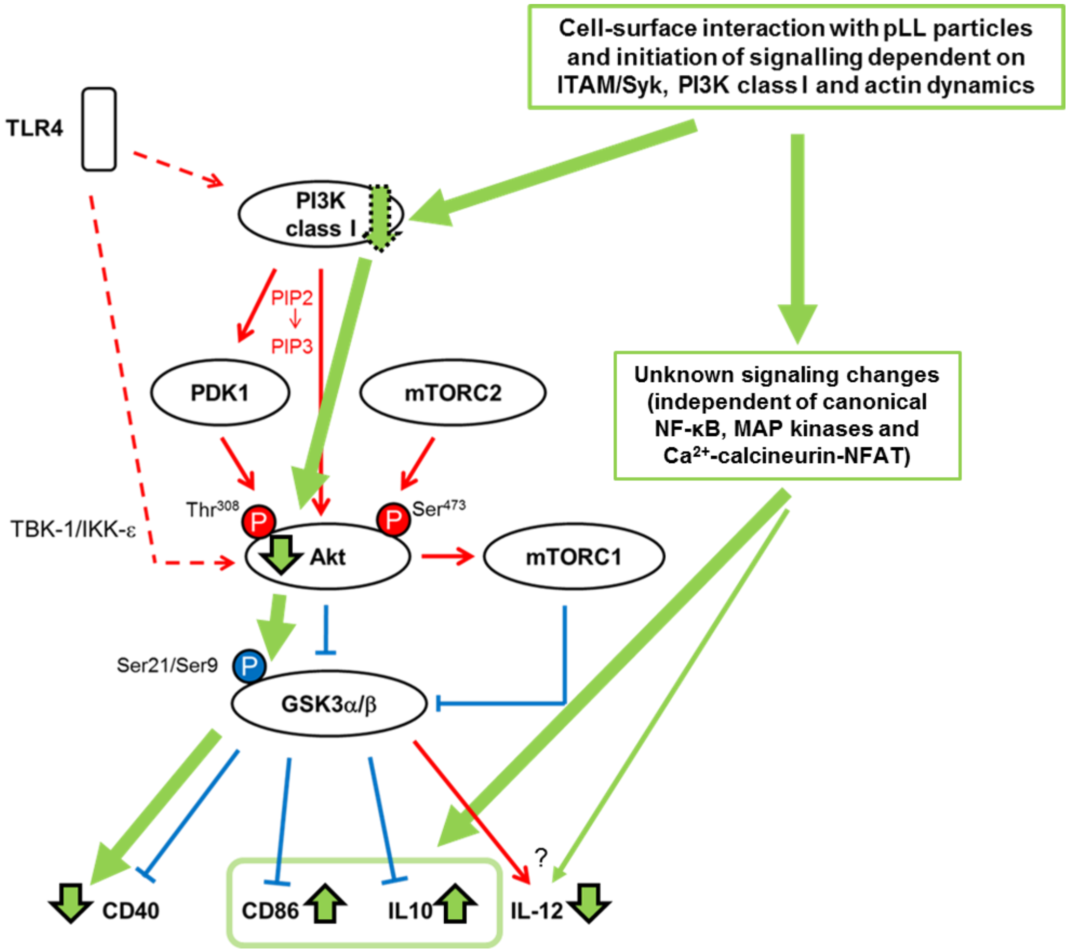

FIG S6. Summary of proposed mechanisms underlying the effects of pLL on GMCSF-BMDCs. The proposed changes brought about by pLL are shown in green, plotted onto the diagram shown in Fig. S1b (which summarizes relevant changes in the PI3K-Akt-GSK3 pathway induced by LPS). Mechanistic links are represented by long arrows without borders, and changes in kinase activity or in protein expression are shown by short arrows with black borders.

The cell-surface interaction and signaling envisaged is similar to membrane affinity-triggered signaling (MATS). MATS requires actin remodeling, PI3K and Syk, although the precise mechanistic relationships between these players have not been elucidated (23-25); the role of Syk in MATS would be independent from its role in LPS-stimulated, CD14-dependent signaling shown in Fig. S1a. We propose that in the context of a MATS-like interaction with pLL particles, receptor-activated PI3K class I activity is diminished, possibly as a consequence of decreased availability of its PIP2 substrate (24, 26).

Diminished PI3K activity in the presence of pLL blunts Akt activation, and hence enhances GSK3 activity, which in turn blunts CD40 up-regulation. Parallel, unidentified signaling changes downstream of the MATS-like interaction, independent of the NF-κB, MAP kinases and Ca^{2+}-calcineurin-NFAT pathways, are responsible for the effects of pLL on CD86 and IL-10; these unidentified changes would overwhelm the opposite effects on CD86 and IL-10 brought about by the enhanced GSK3 activity. The effects of pLL on IL-12 expression would be brought about by Akt-dependent and Akt-independent signaling changes interacting in complex ways that we do not have a model for, as indicated by the question mark in the diagram.

Although not represented in the diagram, the MATS-like interaction with pLL and the blunting of receptor-activated PI3K activity would also arise in the absence of LPS stimulation, explaining that pLL also inhibits Akt activation in response to IL-4 or GM-CSF (27).

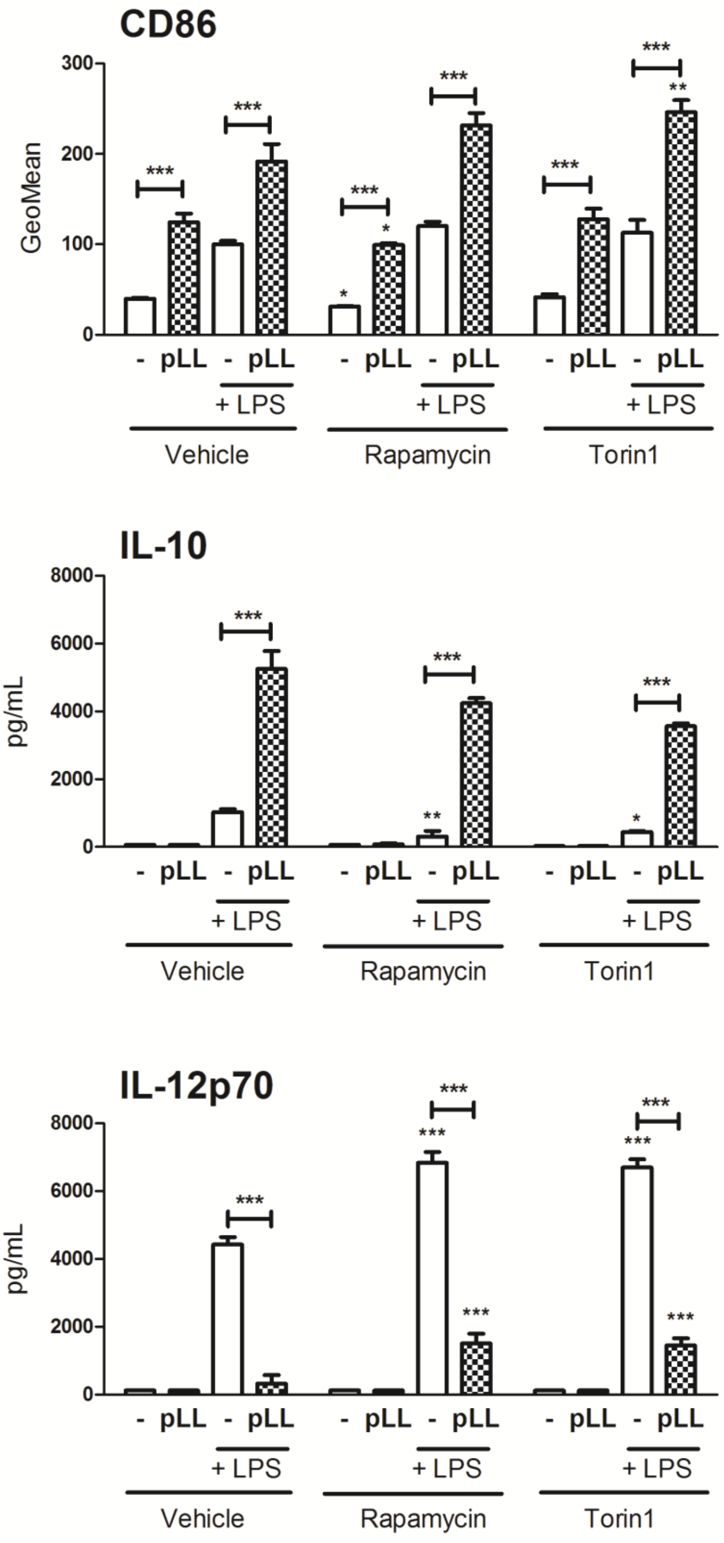

FIG S7 (related to Figs. 2 and 3). Effects of mTORC1 inhibitors on CD86, IL-10 and IL-12 expression in GMCSF-BMDCs, in the absence and presence of pLL. GMCSF-BMDCs were exposed to pLL, LPS, or both stimuli together, for 18 h, in the absence or presence of the mTORC1 inhibitors rapamycin or torin1. Cell surface expression of CD86, and IL-10 and IL-12p70 levels in supernatants were measured. All data shown correspond to means +/- SD of triplicate wells. Results shown are representative of 3 independent experiments.

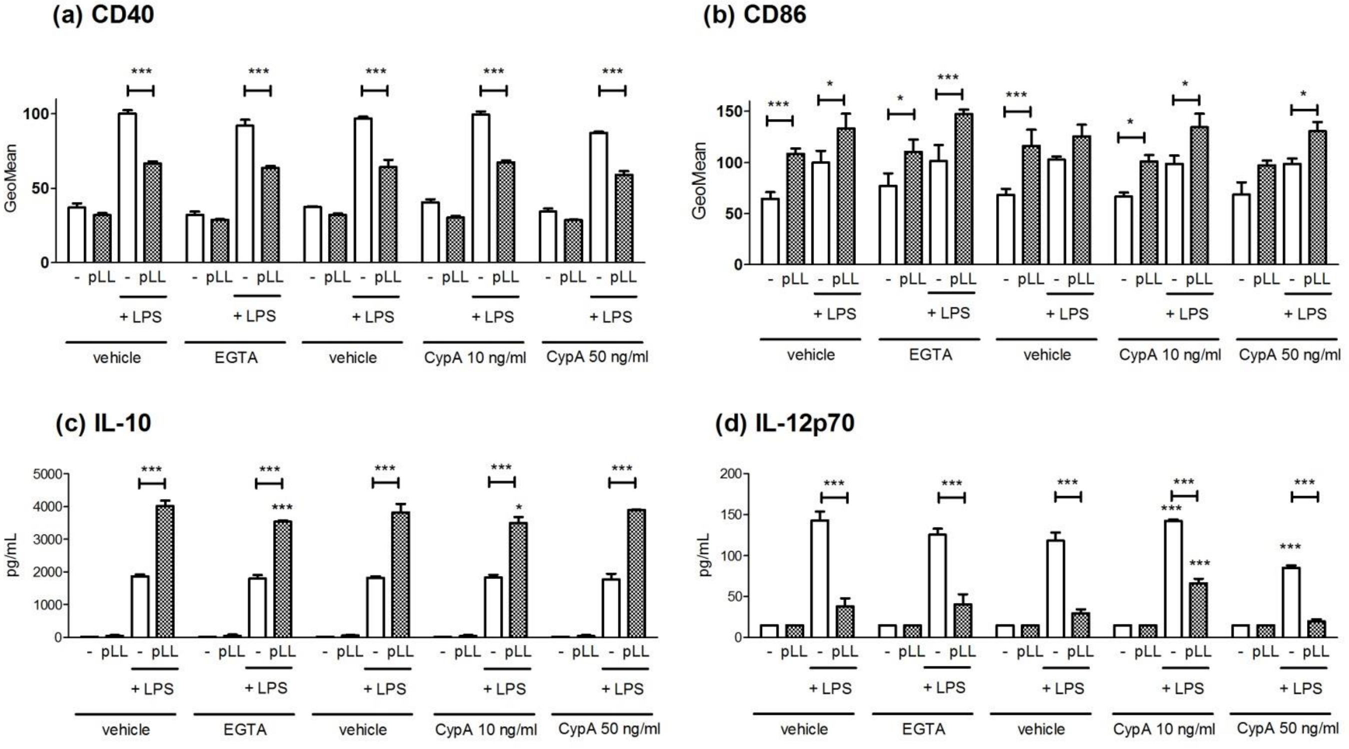

FIG S8. The effects of pLL on GMCSF-BMDCs are not abrogated by inhibitors of the calcium-calcineurin-NFAT pathway. GMCSF-BMDCs were exposed to pLL, LPS, or both stimuli together, for 18 h, in the absence or presence of the extracellular calcium sequestering agent EGTA or the calcineurin inhibitor cyclosporine A at two different concentrations. Cell surface expression of CD40 and CD86 and levels of IL-10 and IL-12p70 in supernatants were measured. All values plotted correspond to means +/- SD of triplicate wells. Results shown are representative of 2 or 3 independent experiments. The small effects of EGTA and cyclosporine A (10 ng/mL) on IL-10 and IL-12p70 production were not reproducible across experiments.

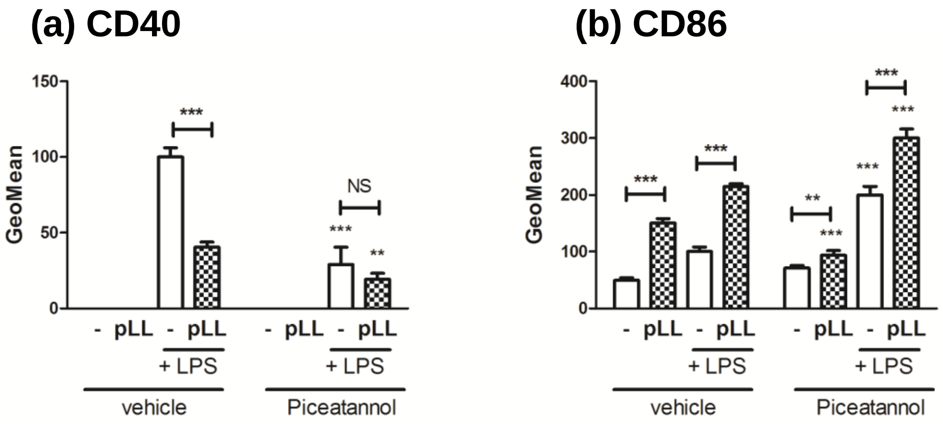

FIG S9. Syk may be involved in the effects of pLL on the phenotype of GMCSF-BMDCs. GMCSF-BMDCs were exposed to pLL, LPS, or both stimuli together, for 18 h, in the absence or presence of the Syk inhibitor piceatannol. Cell surface expression of CD40 (a) and CD86 was measured. All values plotted correspond to means +/- SD of triplicate wells. Results shown are representative of three independent experiments.

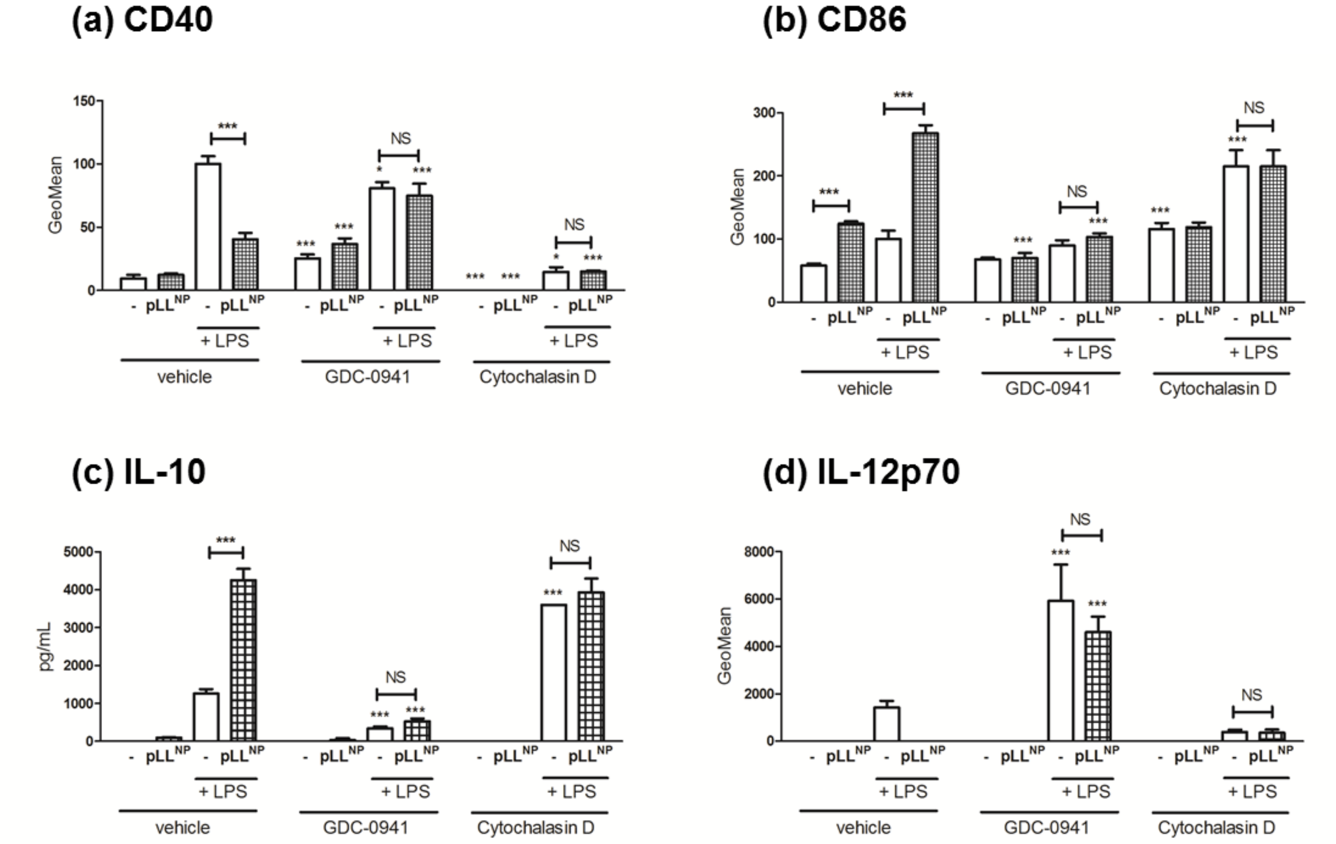

FIG S10 (related to Fig. 7). The phenotypic effects of non-phagocytosable pLL on GMCSF-BMDCs require actin dynamics and functional PI3Ks class I. GMCSF-BMDCs were exposed to pLL selected for non-phagocytosable particle size range (“pLL^{NP}”), LPS, or both stimuli together, for 18 h in the absence and presence of PI3K class I inhibitor (GDC-0941) or actin dynamics inhibitor (cytochalasin D), and cell surface expression of CD40 (a) and CD86 (b), and IL-10 (c) and IL-12p70 (d) levels in supernatants were measured. All data shown correspond to means +/- SD of triplicate wells. Results shown are representative of 2 independent experiments.

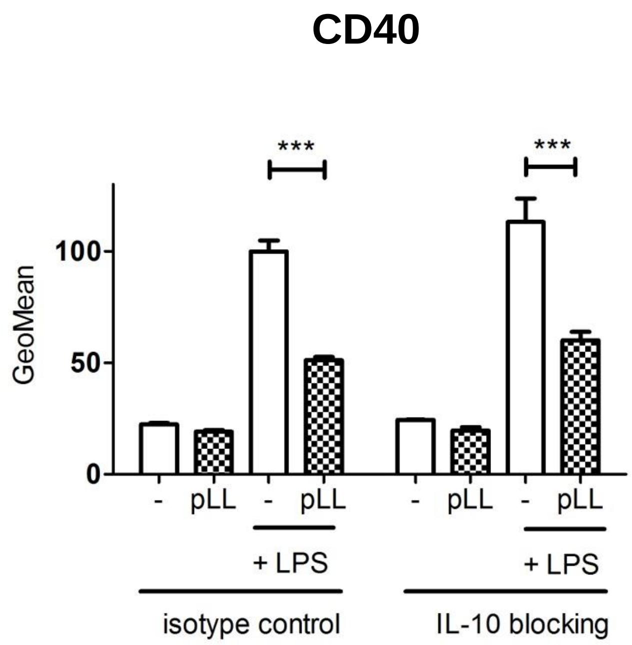

FIG S11. Paracrine IL-10 does not contribute to blunted CD40 up-regulation in GMCSF-BMDCs exposed to pLL and co-stimulated with LPS. GMCSF-BMDCs were exposed to pLL, LPS, or both stimuli together, for 18 h, in presence of either control antibody or an antibody blocking mouse IL-10 (both at 1 µg/mL), and cell surface expression of CD40 was measured. Data shown correspond to means +/- SD of triplicate wells. Results shown are representative of 3 independent experiments.

Respiration (pmol/min.µg protein)	Medium only	pLL	LPS	pLL + LPS
**Basal**	20 ± 2	24 ± 4	4.0 ± 0.8	6 ± 2
**ATP independent**	3.1 ± 0.3	3.9 ± 0.6	1.9 ± 0.5	3 ± 2
**ATP dependent**	17 ± 2	20 ± 3	2.1 ± 0.3	2.4 ± 0.4
**Maximum**	28 ± 2	32 ± 3	0.8 ± 0.4	2 ± 1
**Non-mitochondrial**	5 ± 3	10 ± 1	17 ± 1	16 ± 2

TABLE S1 Mitochondrial respiratory parameters of GMCSF-BMDCs stimulated with pLL and/or LPS. Respiratory parameters were calculated from the data shown in Fig. 4 as described in the Methods section. Results are presented as mean ± SD of triplicate wells.

Target	Supplier	Catalog number	Dilution
IκB-α	Cell Signaling Technology	9242	1:1000
phospho-p38 (Thr^{180}/Tyr^{182})	Cell Signaling Technology	4511	1:1000
phospho-JNK/SAPK (Thr^{183}/Tyr^{185})	Cell Signaling Technology	4668	1:1000
phospho-ERK 1/2 (Thr^{202}/Tyr^{204})	Cell Signaling Technology	4370	1:1000
phospho-Akt (Ser^{473})	Cell Signaling Technology	4060	1:1000
Akt	Cell Signaling Technology	9272	1:1000
phospho-GSK3-α/β (Ser^{21}/Ser^{9})	Cell Signaling Technology	8566	1:1000
GSK3-β	Cell Signaling Technology	12456	1:1000
α-tubulin	Santa Cruz Biotechnology	sc-8035	1:250
β-actin	Cell Signaling Technology	4967	1:1000

Table S2. Antibodies used in Western blotting. The anti- α-tubulin primary antibody is a mouse IgM, and it was detected using a corresponding secondary antibody from Invitrogen (M31507). All the other primary antibodies are rabbit IgG, and were detected using corresponding secondary antibodies from Cell Signaling Technologies (7074) or Calbiochem (401353).

References in Supplementary Materials

1. Rescigno M, Martino M, Sutherland CL, Gold MR, Ricciardi-Castagnoli P. 1998. Dendritic cell survival and maturation are regulated by different signaling pathways. J Exp Med 188:2175-80.
2. Tone M, Tone Y, Babik JM, Lin CY, Waldmann H. 2002. The role of Sp1 and NF-kappa B in regulating CD40 gene expression. J Biol Chem 277:8890-7.
3. Gabrysova L, Howes A, Saraiva M, O'Garra A. 2014. The regulation of IL-10 expression. Curr Top Microbiol Immunol 380:157-90.
4. Kaisho T, Tanaka T. 2008. Turning NF-kappaB and IRFs on and off in DC. Trends Immunol 29:329-36.
5. Qian C, Jiang X, An H, Yu Y, Guo Z, Liu S, Xu H, Cao X. 2006. TLR agonists promote ERK-mediated preferential IL-10 production of regulatory dendritic cells (diffDCs), leading to NK-cell activation. Blood 108:2307-15.
6. Zanoni I, Ostuni R, Capuano G, Collini M, Caccia M, Ronchi AE, Rocchetti M, Mingozzi F, Foti M, Chirico G, Costa B, Zaza A, Ricciardi-Castagnoli P, Granucci F. 2009. CD14 regulates the dendritic cell life cycle after LPS exposure through NFAT activation. Nature 460:264-8.
7. Zanoni I, Ostuni R, Marek LR, Barresi S, Barbalat R, Barton GM, Granucci F, Kagan JC. 2011. CD14 controls the LPS-induced endocytosis of Toll-like receptor 4. Cell 147:868-80.
8. Fric J, Zelante T, Wong AY, Mertes A, Yu HB, Ricciardi-Castagnoli P. 2012. NFAT control of innate immunity. Blood 120:1380-9.
9. Beinke S, Ley SC. 2004. Functions of NF-kappaB1 and NF-kappaB2 in immune cell biology. Biochem J 382:393-409.
10. Rodionova E, Conzelmann M, Maraskovsky E, Hess M, Kirsch M, Giese T, Ho AD, Zoller M, Dreger P, Luft T. 2007. GSK-3 mediates differentiation and activation of proinflammatory dendritic cells. Blood 109:1584-92.
11. Martin M, Rehani K, Jope RS, Michalek SM. 2005. Toll-like receptor-mediated cytokine production is differentially regulated by glycogen synthase kinase 3. Nat Immunol 6:777-84.
12. Ohtani M, Nagai S, Kondo S, Mizuno S, Nakamura K, Tanabe M, Takeuchi T, Matsuda S, Koyasu S. 2008. Mammalian target of rapamycin and glycogen synthase kinase 3 differentially regulate lipopolysaccharide-induced interleukin-12 production in dendritic cells. Blood 112:635-43.
13. Alessandrini A, De Haseth S, Fray M, Miyajima M, Colvin RB, Williams WW, Benedict Cosimi A, Benichou G. 2011. Dendritic cell maturation occurs through the inhibition of GSK-3beta. Cell Immunol 270:114-25.
14. Park D, Lapteva N, Seethammagari M, Slawin KM, Spencer DM. 2006. An essential role for Akt1 in dendritic cell function and tumor immunotherapy. Nat Biotechnol 24:1581-90.
15. Wang B, Liu TY, Lai CH, Rao YH, Choi MC, Chi JT, Dai JW, Rathmell JC, Yao TP. 2014. Glycolysis-dependent histone deacetylase 4 degradation regulates inflammatory cytokine production. Mol Biol Cell 25:3300-7.
16. Wang H, Brown J, Garcia CA, Tang Y, Benakanakere MR, Greenway T, Alard P, Kinane DF, Martin M. 2011. The role of glycogen synthase kinase 3 in regulating IFN-beta-mediated IL-10 production. J Immunol 186:675-84.
17. Wang H, Brown J, Gu Z, Garcia CA, Liang R, Alard P, Beurel E, Jope RS, Greenway T, Martin M. 2011. Convergence of the mammalian target of rapamycin complex 1- and glycogen synthase kinase 3-beta-signaling pathways regulates the innate inflammatory response. J Immunol 186:5217-26.
18. Woodgett JR, Ohashi PS. 2005. GSK3: an in-Toll-erant protein kinase? Nat Immunol 6:751-2.
19. Troutman TD, Bazan JF, Pasare C. 2012. Toll-like receptors, signaling adapters and regulation of the pro-inflammatory response by PI3K. Cell Cycle 11:3559-67.
20. Pittini Á, Casaravilla C, Allen JE, Díaz Á. 2016. Pharmacological inhibition of PI3K class III enhances the production of pro- and anti-inflammatory cytokines in dendritic cells stimulated by TLR agonists. Int Immunopharmacol 36:213-217.
21. Hawkins PT, Stephens LR. 2015. PI3K signalling in inflammation. Biochim Biophys Acta 1851:882-97.
22. Everts B, Amiel E, Huang SC, Smith AM, Chang CH, Lam WY, Redmann V, Freitas TC, Blagih J, van der Windt GJ, Artyomov MN, Jones RG, Pearce EL, Pearce EJ. 2014. TLR-driven early glycolytic reprogramming via the kinases TBK1-IKK-repsilon supports the anabolic demands of dendritic cell activation. Nat Immunol 15:323-32.
23. Flach TL, Ng G, Hari A, Desrosiers MD, Zhang P, Ward SM, Seamone ME, Vilaysane A, Mucsi AD, Fong Y, Prenner E, Ling CC, Tschopp J, Muruve DA, Amrein MW, Shi Y. 2011. Alum interaction with dendritic cell membrane lipids is essential for its adjuvanticity. Nat Med 17:479-87.
24. Mu L, Tu Z, Miao L, Ruan H, Kang N, Hei Y, Chen J, Wei W, Gong F, Wang B, Du Y, Ma G, Amerein MW, Xia T, Shi Y. 2018. A phosphatidylinositol 4,5-bisphosphate redistribution-based sensing mechanism initiates a phagocytosis programing. Nat Commun 9:4259.
25. Ng G, Sharma K, Ward SM, Desrosiers MD, Stephens LA, Schoel WM, Li T, Lowell CA, Ling CC, Amrein MW, Shi Y. 2008. Receptor-independent, direct membrane binding leads to cell-surface lipid sorting and Syk kinase activation in dendritic cells. Immunity 29:807-18.
26. Saito K, Tolias KF, Saci A, Koon HB, Humphries LA, Scharenberg A, Rawlings DJ, Kinet JP, Carpenter CL. 2003. BTK regulates PtdIns-4,5-P2 synthesis: importance for calcium signaling and PI3K activity. Immunity 19:669-78.
27. Seoane PI, Rückerl D, Casaravilla C, Barrios AA, Pittini A, MacDonald AS, Allen JE, Díaz A. 2016. Particles from the *Echinococcus granulosus* laminated layer inhibit IL-4 and growth factor-driven Akt phosphorylation and proliferative responses in macrophages. Sci Rep 6:39204.
